# ARGNet: using deep neural networks for robust identification and classification of antibiotic resistance genes from sequences

**DOI:** 10.1186/s40168-024-01805-0

**Published:** 2024-05-09

**Authors:** Yao Pei, Marcus Ho-Hin Shum, Yunshi Liao, Vivian W. Leung, Yu-Nong Gong, David K. Smith, Xiaole Yin, Yi Guan, Ruibang Luo, Tong Zhang, Tommy Tsan-Yuk Lam

**Affiliations:** 1 State Key Laboratory of Emerging Infectious Diseases, School of Public Health, The University of Hong Kong, Pokfulam, Hong Kong SAR, China; 2 Joint Institute of Virology (Shantou University and The University of Hong Kong), Guangdong-Hongkong Joint Laboratory of Emerging Infectious Diseases, Shantou University, Shantou, Guangdong 515063 China; 3 Laboratory of Data Discovery for Health (D²4H), Hong Kong Science Park, Pak Shek Kok, Hong Kong SAR, China; 4 Advanced Pathogen Research Institute, Futian District, Shenzhen City, Guangdong 518045 China; 5 Centre for Immunology & Infection (C2i), Hong Kong Science Park, Pak Shek Kok, Hong Kong SAR, China; 6 Division of Biotechnology, Research Center of Emerging Viral Infections, College of Medicine, Chang Gung University, Taoyuan, Taiwan; 7 International Master Degree Program for Molecular Medicine in Emerging Viral Infections, College of Medicine, Chang Gung University, Taoyuan, Taiwan; 8 Department of Laboratory Medicine, Linkou Chang Gung Memorial Hospital, Taoyuan, Taiwan; 9 National Institute of Infectious Diseases and Vaccinology, National Health Research Institutes, Zhunan, Taiwan; 10 Department of Civil Engineering, The University of Hong Kong, Pokfulam, Hong Kong SAR, China; 11 Department of Computer Science, The University of Hong Kong, Pokfulam, Hong Kong SAR, China

**Keywords:** Antibiotic resistance, Antibiotic resistance genes, Deep learning, Autoencoder, Multiclass classification convolutional neural network, ARGNet

## Abstract

**Background:**

Emergence of antibiotic resistance in bacteria is an important threat to global health. Antibiotic resistance genes (ARGs) are some of the key components to define bacterial resistance and their spread in different environments. Identification of ARGs, particularly from high-throughput sequencing data of the specimens, is the state-of-the-art method for comprehensively monitoring their spread and evolution. Current computational methods to identify ARGs mainly rely on alignment-based sequence similarities with known ARGs. Such approaches are limited by choice of reference databases and may potentially miss novel ARGs. The similarity thresholds are usually simple and could not accommodate variations across different gene families and regions. It is also difficult to scale up when sequence data are increasing.

**Results:**

In this study, we developed ARGNet, a deep neural network that incorporates an unsupervised learning autoencoder model to identify ARGs and a multiclass classification convolutional neural network to classify ARGs that do not depend on sequence alignment. This approach enables a more efficient discovery of both known and novel ARGs. ARGNet accepts both amino acid and nucleotide sequences of variable lengths, from partial (30–50 aa; 100–150 nt) sequences to full-length protein or genes, allowing its application in both target sequencing and metagenomic sequencing. Our performance evaluation showed that ARGNet outperformed other deep learning models including DeepARG and HMD-ARG in most of the application scenarios especially quasi-negative test and the analysis of prediction consistency with phylogenetic tree. ARGNet has a reduced inference runtime by up to 57% relative to DeepARG.

**Conclusions:**

ARGNet is flexible, efficient, and accurate at predicting a broad range of ARGs from the sequencing data. ARGNet is freely available at https://github.com/id-bioinfo/ARGNet, with an online service provided at https://ARGNet.hku.hk.

Video Abstract

**Supplementary Information:**

The online version contains supplementary material available at 10.1186/s40168-024-01805-0.

## Background

Antibiotics are an essential clinical foundation for treating most bacterial infections. However, worldwide, effectiveness of antibiotics is limited by resistance among bacteria to such drugs. Bacterial resistance to antibiotics can arise through genetic mutations or horizontal gene transfer among bacteria. High usage of antibiotics is a major evolutionary driver of antibiotic resistance, which is now considered a global public health threat [1, 2]. Given the global scope and microbiological complexity of resistance patterns and their underlying genes, more effective and efficient methods to track its emergence and prevalence are needed.

Antimicrobial susceptibility testing (AST), in which bacteria are cultivated in vitro and then exposed specific antibiotics to determine sensitivity or resistance patterns, is the standard method for determining resistance patterns among bacteria. It is relatively slow, low-throughput, and can be biased and limited only to culturable microbes [3, 4]. Alternative methods are next-generation sequencing (NGS) technologies and their related computational capabilities that allow sequence-based methods to detect antibiotic resistance genes (ARGs) [5]. These technologies extend to metagenomic analyses that enable the discovery of novel ARGs, identification of their global distribution, and the tracking of multidrug-resistant bacteria in clinical and natural environments [6–9].

Bacterial DNA can also be extracted from environmental samples (e.g., water, soil) and sequenced using metagenomics approaches. ARGs in such samples usually are identified by comparing sample sequences against those in reference databases. Short reads from a sequencing platform, or assembled contigs (contiguous fragments), can be annotated with resistance labels by sequence similarity-based alignment tools (such as BLAST [10], DIAMOND [11], and Bowtie [12]). These methods are limited by using fixed, usually high, cutoffs for global sequence identity (possibly 80% or 90%), which may lead to a high false-negative rate [13, 14]. And it is difficult to find out a universal cutoff optimal for different ARG categories. The accuracy of a sequence alignment depends on the underlying sequence similarity between the query and the reference sequences, while two unrelated protein sequences can match at up to 25% residues if gaps are allowed [15]. Alignment-based methods do not scale well with increasing sequence length and numbers [15].

Deep learning has been successfully deployed in many bioinformatics applications [16–20]. It allows a feature to be learnt directly from the data using a general-purpose procedure. DeepARG [21] and HMD-ARG [22] are deep learning models developed to identify ARGs. DeepARG detects ARGs from metagenomics data using sequence similarity scores calculated from BLAST against reference database to train the network. It still inherits the disadvantages of alignment-based methods and does not consider sub-sequence patterns such as genomic changes or regulatory elements beyond similarity [21]. The model scalability also suffers from increasing amounts of data and reference sequences. HMD-ARG [22] only works on amino acid sequences, which must conform to a limited range of lengths (50–1571 amino acids). Its performance on shorter contigs or short reads is not well studied.

Here, we developed a computational tool, denoted as ARGNet, based on deep neural networks to identify ARGs of variable lengths and classify them into 36 categories of antibiotics resistance. ARGNet-L was built to predict long sequences and ARGNet-S for short sequences. Both models have the same architecture: an autoencoder network followed by a convolutional neural network (CNN). ARGNet embeds sequences into a latent space using an unsupervised learning autoencoder neural network, so that ARGs are better reconstructed than non-ARGs. The following CNN predicts the category of antibiotic that the ARG resists. ARGNet was designed for four types of data input including nucleotide sequences and their translated amino acids that are either short reads directly generated from metagenomic short (100–150 nucleotides or 30–50 amino acids) or long (greater than 150 nucleotides or 50 amino acids) sequences that are generated by target sequencing or assembled contigs from NGS reads, carrying partial or complete genes (full-length sequences or contigs with different lengths). ARGNet outperformed current deep learning models, which have different model frameworks, on multiple datasets.

## Methods

### ARGNet-DB

**Fig. 1:**
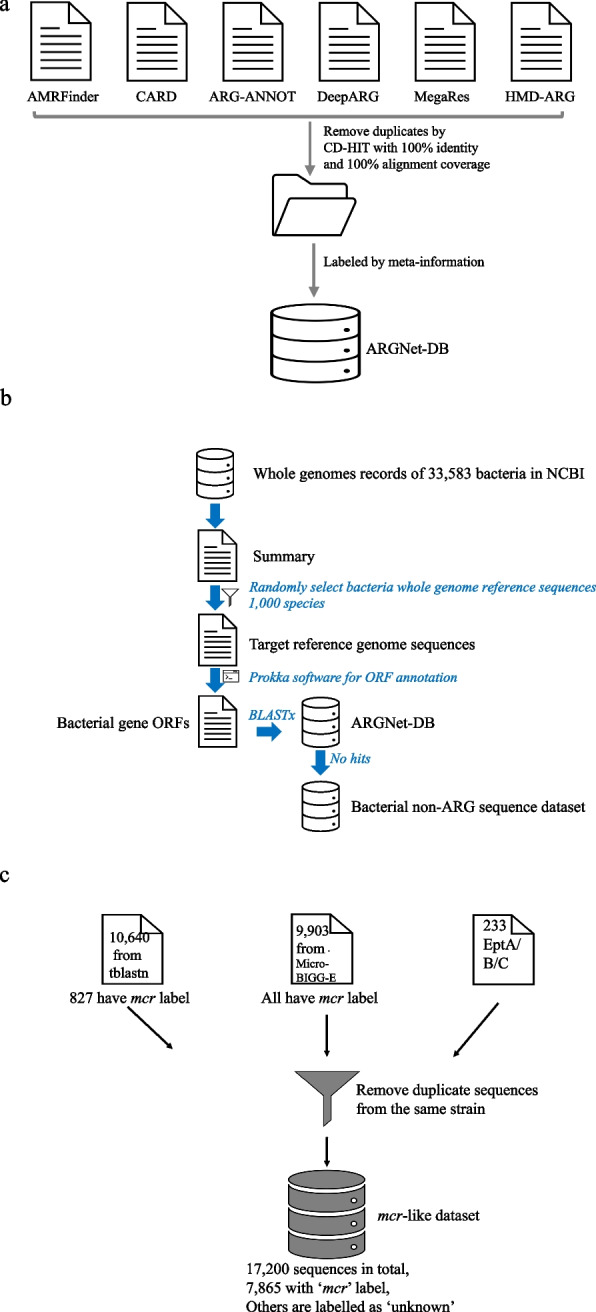
The cleaning and curation process of ARGNet-DB, bacterial non-ARG sequence dataset, and mcr-like dataset. **a** Data collection and curation for ARGNet-DB. ARG sequences were collected from six major public databases followed by removal of duplicate sequences and annotation from metadata. **b** Data collection and manipulation of bacterial non-ARG sequence dataset. **c** Data collection and de-duplication for *mcr* test dataset

Antibiotic resistance gene sequences were collected from six major databases, CARD (v 3.1.2) [23], AMRFinder [24], ResFinder [25], Megares [26], deepARG [21], and HMD-ARG [22], composed of 48,615 amino acid sequences. Sequences annotated as conferring resistance by single-nucleotide polymorphisms (SNPs) were removed. CD-HIT [27], with settings of 100% sequence identity and full-length alignment coverage, was used to remove duplicate sequences, leaving 27,464 unique ARG sequences. Sequences were labelled by the antibiotic resistance retrieved from metadata in the source databases (Fig. 1a). Sequences exhibiting resistance to multiple categories of drugs are appropriately annotated with the “multidrug” label. These sequences were assigned into 36 antibiotic resistance categories (Fig. 3a). In terms of model implementation, 80% of the data was randomly selected as the training set, and the remaining 20% (ARG-test-db) was used as the test set.

### Data encoding

Input (amino acid) sequences for the deep learning model were encoded to a matrix format by the one-hot method. For a sequence $X$, any single position ${X}_{i,j}$ was encoded to a one-hot value as follows:

$$X_{i,j}=\left\{\begin{array}{c}1\;\mathrm{if}\;{\mathrm s}_i={\mathrm c}_j\\0.5\;\mathrm{if}\;{\mathrm s}_i=B\;\mathrm{and}\;c_j\in\left\{D,N\right\}\\\mathrm{or}\;{\mathrm s}_i=Z\;\mathrm{and}\;c_j\in\left\{\text{E},\text{Q}\right\}\\\mathrm{or}\;{\mathrm s}_i=\mathrm J\;\mathrm{and}\;{\mathrm c}_j\in\left\{\text{I},\text{L}\right\}\\0\;\mathrm{otherwise}\end{array}\right.$$

where ${s}_{i}$ was the $i$th position in the sequence and ${c}_{j}$ was the ${\text{j}}$th code in the code set, which comprised the 20 single-character amino acid codes. Characters $B$, $Z$, and $J$ are ambiguity codes: $B=D\ or\ N$, $Z=E\ or\ Q$, and $J= I\ or\ L$.

### Model implementation

The model was built with Python3 and Keras, which is an interface to the TensorFlow library as the deep learning framework. For both ARGNet-S and ARGNet-L, the training dataset was first one-hot encoded and then input into the autoencoder to filter the ARG sequences, which were passed to the CNN classifier to predict the category of antibiotic resistance.

After hundreds to thousands of experiments, the best-performing models of ARGNet-S and ARGNet-L were selected and are described below.

In ARGNet-S, the autoencoder model contained 35 one-dimensional convolutional layers (14 in the encoder and 21 in the decoder) and 4 one-dimensional max-pooling layers, each with a kernel size of 2, in the encoder and 4 one-dimensional upsampling layers in the decoder. The CNN classifier contained four convolutional layers and five dense layers and an attention mechanism to address positive features learnt by the previous convolutional layers.

In ARGNet-L, the autoencoder had 28 one-dimensional convolutional layers (14 in the encoder and 14 in the decoder) and 6 max pooling layers in both the encoder and decoder. The CNN classifier contained four one-dimensional convolutional layers, two dense layers, and an attention mechanism.

Details on the filter numbers in each convolutional and dense layer, the learning rate of each model, and other hyperparameters or parameters are in the Additional file 1: Tables S1–4. For both the autoencoder and CNN classifier models, an augmentation operation was performed on the training data. For ARGNet-L, the autoencoder and CNN classifier were trained in mini batches mixed with full-length sequences and 90%, 80%, 70%, and 60% of full-length sequences. For ARGNet-S, both the autoencoder and CNN classifier were trained with mini batches that were mixed with 30–50 amino acid sequences.

The model is provided for use in local machines (https://github.com/id-bioinfo/ARGNet) and can be used through the online analysis platform (https://ARGNet.hku.hk).

### Test datasets

Four datasets were used for testing, each of which was manipulated to be evaluated in different application scenarios. To test ARGNet-L with long sequences, five types of (sub) sequences (both amino acid, LSaa, and nucleotide, LSnt) were generated for each sequences in each test dataset. They were the full-length sequences, and randomly generated subsequences with 90%, 80%, 70%, and 60% of full-length sequences, attempting to simulate applications containing both full-length sequences and contigs of different lengths. To test ARGNet-S with short sequences, three amino acid test datasets (SSaa) of 30, 40, and 50 amino acids and three nucleotide test datasets (SSnt) consisting of 100, 120, and 150 nucleotides were randomly sub-sequenced five times from each full-length test sequences.

**Fig. 2:**
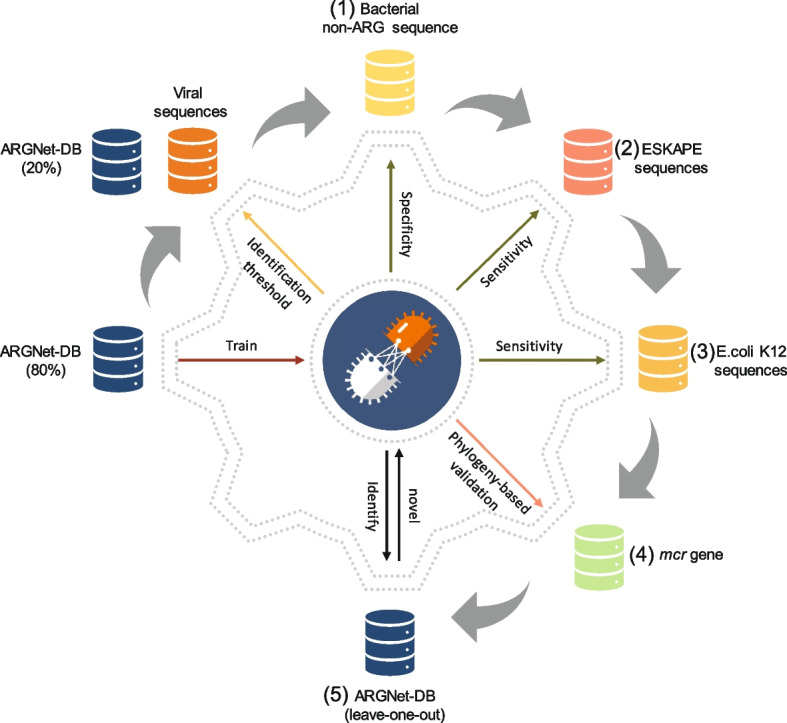
Illustration of testing and validation experiments conducted in this study

The testing and validation experiments conducted in this study are outlined in Fig. 2. To establish a threshold for distinguishing between ARGs and non-ARGs, an initial experiment was performed using an ARG test dataset and a virus test dataset. Furthermore, five additional experiments were conducted to assess the performance of ARGNet under various scenarios:

1. Bacterial non-ARG sequences were curated to evaluate the threshold derived from the ARG and virus sequences. These bacterial non-ARG sequences, being more closely related to ARGs than viruses, allowed us to test the model’s specificity in identifying non-ARGs based on the threshold defined using viruses as negative data.
2. Sequences from ESKAPE pathogens, ranging from 0 to 60% identity (providing less contrast and intermediate hits) [28] to ARGNet-DB, were collected to further evaluate the sensitivity of ARGNet towards the primary sources of well-characterized ARGs.
3. Sequences (protein) from *Escherichia coli* K12 [29] were also curated to assess the sensitivity of ARGNet towards one of the most extensively studied microorganisms.
4. The *mcr* gene sequences were collected to investigate the consistency of resistance phenotype prediction between ARGNet and phylogenetic tree inference*.*
5. A quasi-negative test, based on ARGNet-DB, was conducted to evaluate the performance of ARGNet in detecting novel ARGs.

The detailed process of preparing each test dataset is described in the subsequent sections.

### ARG test dataset

Twenty percent of ARGNet-DB were used as positive test samples (ARG-test-db, 5490 sequences).

### Virus test dataset

Virus sequences were used as negative dataset. A total of 4983 protein sequences from 12 animal virus families were downloaded from the NCBI Protein RefSeq Database (accession date: 28 June 2021). These families were Arenaviridae (198 sequences), Coronaviridae (595), Filoviridae (100), Flaviviridae (194), Hantaviridae (137), Orthomyxoviridae (164), Paramyxoviridae (534), Phenuiviridae (412), Picornaviridae (178), Pneumoviridae (95), Reoviridae (1040), and Rhabdoviridae (1336).

### Bacterial non-ARG sequence dataset

Whole genome records were available for 33,583 bacteria species in NCBI by June 2022. A total of 1000 bacteria species were randomly selected and their reference genome sequences were downloaded. Prokka [30] was used to predict the open reading frames (ORFs) of each genome, which were submitted for BLASTx analysis against ARGNet-DB. ORFs with no hits to ARGNet-DB were retained as the bacterial non-ARG sequence dataset to validate the identification threshold (Fig. 1b).

### ESKAPE dataset

Full genomic reference sequences of ESKAPE were downloaded from NCBI via NCBI Datasets tool (accessed by Dec 18, 2023). To construct the ESKAPE test dataset, 10 GCF files for each ESKAPE pathogen were randomly selected, and the corresponding “protein.faa” files were extracted, resulting in a total of 250,039 protein sequences to comprise the test dataset.

### *E. coli* K12 dataset

Sixty-six full genomes of *E. coli* K12 were downloaded (accessed by Dec 18, 2023), and after removing duplicated sequences, a total of 5349 protein sequences were extracted to serve as the test dataset. A total of 393 proteins were designated as transporters based on their NCBI annotation.

### mcr-like dataset

Two distantly related *mcr* RefSeq protein sequences (*mcr-*1, WP_163397051.1; *mcr-*4, WP_099156046.1) were used as query sequences for a tBLASTn search against the NCBI nucleotide database. The top 5000 results with the lowest *e*-value (in ascending order) in each tBLASTn search (resulting in 10,640 aligned sequences) were defined as *mcr*-like sequences (as to capture a large number of *mcr*-like sequences). Another set of 9903 *mcr*-labelled genes were obtained from the NCBI MicroBIGG-E web service (https://www.ncbi.nlm.nih.gov/pathogens/microbigge/). These two datasets were merged and de-duplicated, and 16,967 sequences were retained. In addition, 233 *EptA/B/C* (*mcr*-like) sequences from one comparative study [31] were also included (no duplication with the cleaned 16,967 sequences). The final *mcr*-like dataset contained 17,200 sequences (Fig. 1c).

### Phylogenetic tree implementation

To deduce the antibiotics resistance of the *mcr-like* dataset, altogether, 17,200 sequences were aligned using FFT-NS-i method from MAFFT [32]. Maximum likelihood phylogenetic tree was reconstructed as implemented in FastTree [33]. A total of 7865 sequences were labelled as “mcr” from their metadata, and the others were labelled as “unknown.” According to the inferred phylogenetic tree, the definition of *mcr* sequences was expanded. The most recent common ancestor (MRCA) was achieved for each set of *mcr*-1 to *mcr*-10 labelled sequences, and sequences belong to the clade of each MRCA were incorporated into the expanded “mcr” dataset (subtrees of each clade are in Additional file 1: Figs. S1–8). The prediction of three deep learning models was evaluated using the expanded dataset derived from the phylogenetic tree. Model testing followed the standard procedure of model implementation.

### Quasi-negative test implementation

The quasi-negative test was carried out on long amino acid dataset, in a similar way as leave-one-out cross-validation. An entire single category of ARG sequences was selected as quasi-negative data and used exclusively for model testing but not training. The remaining 35 categories were used for model training. Model training and testing followed the procedure of model implementation mentioned above. Quasi-negative test was performed for each of 36 categories, in both ARGNet and DeepARG, resulting into a total of 72 models trained and tested. The performance of those ARGNet and DeepARG models was compared for their capability to identify and classify “novel” category of ARG sequences (i.e., quasi-negative sequences in each leave-out dataset).

### Evaluation metrics

To evaluate the model performance on ARG identification, precision, recall, and F1 score were calculated based on the numbers of true positive (TP), true negative (TN), false positive (FP), and false negative (FN).

Precision is calculated as follows:

$${\text{Precision}}=\frac{TP}{TP+FP}$$

Recall is calculated as follows:

$${\text{Recall}}=\frac{TP}{TP+FN}$$

F1 score is calculated as follows:

$$F1 =\frac{2\times {\text{precision}}\times {\text{recall}}}{{\text{Precision}}+{\text{recall}}}$$

F1 score is the harmonic mean of precision and recall, providing a concordant measurement of model performance.

Classification accuracy of the CNN classifier is defined as the number of correct classifications (with prediction probability > 0.8) divided by the total number of classifications.

Identification specificity during the validation through bacterial non-ARG sequence dataset is defined as the number of identifications as non-ARG divided by total number of non-ARG bacterial sequences.

Prediction accuracy of *mcr* dataset is defined as the number of correct predictions divided by the total number of *mcr* sequences (after expansion).

Sensitivity of quasi-negative test is defined as the number of correct identifications divided by the total number of sequences from quasi-negative category.

### Comparison with other deep learning models

Two other deep learning models, DeepARG and HMD-ARG, were also applied to the same test data (as long as applicable), to compare their performance, prediction accuracy, and runtime, with ARGNet.

A new DeepARG model was trained and tested with the same dataset as ARGNet. For full-length test dataset, the sequence similarity filter (“–iden 80”) and coverage (“–coverage 0.8”) were used. For other subsequence test datasets, sequence similarity was set to the same, while the “coverage” was set according to their proportion to full-length sequences, namely, 0.72, 0.64, 0.56, and 0.48, respectively. For short read test dataset, the “–iden 80” was used for sequence similarity, and coverage was set to 24, 32, and 40 for 30, 40, and 50 amino acids, respectively. The website service of HMD-ARG (http://www.cbrc.kaust.edu.sa/HMDARG/) was used because the source code of the model was not released. Model testing was performed on long amino acid sequences as it only allows protein sequences ranging from 50 to 1571 amino acids as query.

### Comparison with best hit approach

The test was conducted with the Resistance Gene Identifier (RGI) program [34] by using the ARG test dataset and virus test dataset. These tests were carried out through the RGI web portal on the CARD website. The program was evaluated using two criteria: “Perfect and Strict hits only” and “Perfect, Strict, and Loose hits.” A Perfect RGI match is 100% identical to the reference protein sequence along its entire length, a Strict RGI match is not identical, but the bit score of the matched sequence is greater than the curated BLASTP bit-score cutoff, and loose RGI matches have a bit score less than the curated BLASTP bit-score cutoff. The outcomes of these tests were labelled as RGI_strict and RGI_loose, respectively.

## Results

### The ARGNet-DB

**Fig. 3:**
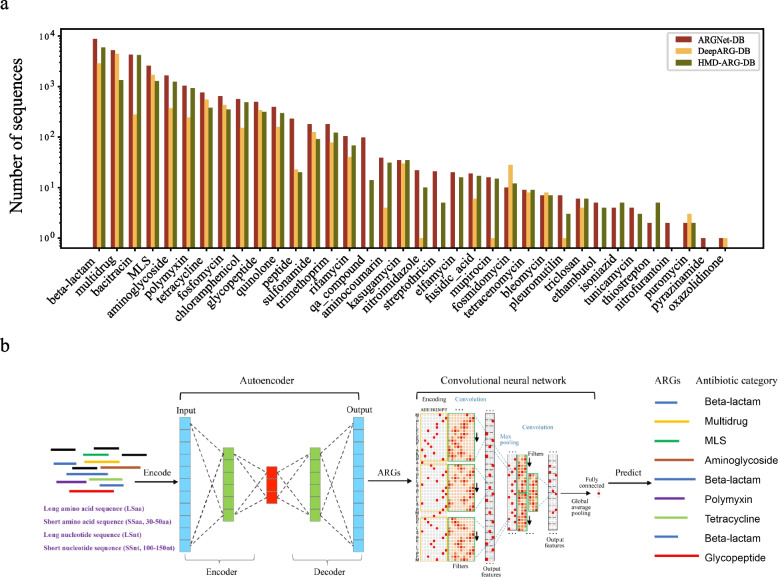
ARGNet-DB and workflow of ARGNet. **a** Composition of ARGNet-DB, number of sequences for 36 ARG categories cleaned in ARGNet-DB (red bar), DeepARG-DB (yellow bar), and HMD-ARG-DB (green bar). *Y*-axis is in log scale. **b** The workflow of ARGNet. Sequences are encoded and passed into the autoencoder. Sequences predicted as ARGs are passed to the convolutional neural network to classify the category of antibiotic they resist

A total of 27,464 ARG amino acid sequences were collected and curated (see “Methods”) to the ARGNet-DB (Fig. 3a) for model training and testing. Sequences in the ARGNet-DB were categorized into 36 antibiotic resistance categories, each of which was updated from 6 ARG public databases (by the middle of year 2021) with experiment support and expert curation. The top three resistance categories with the most sequences in ARGNet-DB were beta-lactam (8810, 32.1%), multidrug (5215, 19%), and bacitracin (4262, 15.5%). Comparatively, there were 28 and 33 ARG categories in databases of DeepARG (DeepARG-DB, 12,279 sequences) and HMD-ARG (HMD-ARG-DB, 17,282), respectively (Fig. 3a). Among the 36 categories, 26 categories are found in all three databases. Streptothricin, qa_compound, elfamycin, ethambutol, isoniazid, tunicamycin, and thiostrepton were curated in ARGNet-DB and HMD-ARG-DB but not DeepARG-DB. Oxazolidinone was curated in ARGNet-DB and DeepARG-DB but not HMD-ARG-DB. Nitrofurantoin and pyrazinamide were curated exclusively in ARGNet-DB.

### The ARGNet

**Fig. 4:**
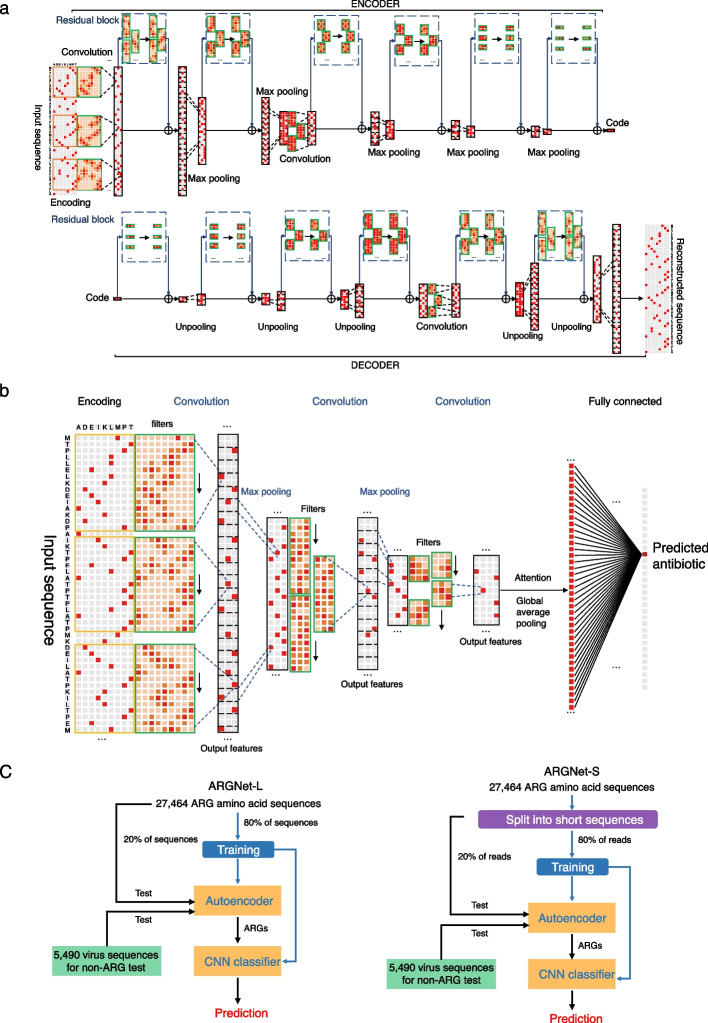
Structure detail of autoencoder and convolutional neural network and experimental workflow. **a** Structure of the autoencoder in ARGNet. Upper part, encoder; lower part, decoder. Major steps are annotated. **b** Structure of the convolutional neural network in ARGNet. **c** training and testing process for ARGNet-L (long sequences, left) and ARGNet-S (short sequences, right)

ARGNet is a two-stage deep neural network. In the first stage, an autoencoder model was developed for identification of ARGs from the input genomic sequence(s). In the second stage, a multiclass CNN was proposed to predict the categories of ARGs from genomic sequences identified as ARGs in the autoencoder model. The input sequences can be long (full length or contigs) or short (30–50 amino acids or 100–150 nucleotides) amino acid or nucleotide sequences. Overview of ARGNet workflow is illustrated in Fig. 3b. The architecture details of autoencoder and CNN in ARGNet are shown in Fig. 4a and b, respectively.

The autoencoder model is an unsupervised artificial neural network designed to learn a data encoding efficiently [35]. An unsupervised model was used here as only ARG sequences (positive samples) were in the database where no sequences were labelled as “non-ARG” (negative samples). The autoencoder had an encoder and a decoder; the encoding (reduction) side is executed, while the decoding (reconstruction) is learnt. In the encoding process, the most representative information of the input ($I$) is compressed by the encoder, and the learnt information is stored in a variable called a latent space (“Code” in Fig. 4a). In the decoding process, the decoder generated output (${I}^{\prime}$) from the latent space that was as close as possible to its original input [36]. In this study, the anomaly detection function of autoencoder was implemented under the assumption that the model will reconstruct ARGs much better than “non-ARGs” as it was only trained with ARGs. ResBlock was built in autoencoder model to make the network deeper to get more ARG-related representation and avoid vanishing or exploding gradients [37]. One-dimensional convolution layers were used as the main neural network layers in the autoencoder and were followed by a batch normalization layer. To identify ARG sequences, a fixed value of reconstruction error was required as identification threshold, which cannot be obtained without negative samples. Thus, a representative collection of virus sequences was incorporated as they are fundamentally “non-ARG.” The threshold was selected such that the total amount of false identification in ARG and “non-ARG” (virus) datasets was minimized.

In the second stage, a CNN was designed to predict the antibiotic resistance category for sequences determined as ARGs by the autoencoder. Filters in convolution layer can be thought as a series of motif or feature detectors (Additional file 1: Table S5, Fig. S10) [38]. The CNN classifier contains convolution layers, followed by max pooling layers with an attention operation to increase the weights of positive output features that are fed into a dense layer to predict the ARG category.

ARGNet was implemented as ARGNet-L and ARGNet-S for long and short sequence inputs, respectively. They both contained an autoencoder for filtering and a CNN for classification. The experimental workflow is shown in Fig. 4c.

### Prediction of long sequences

ARGNet-L was designed to predict whether long sequence (LS; full length or contigs) was ARGs and the resistance category (if it was an ARG). The test dataset contained ARG-test-db (“Methods”) and negative sequence datasets of both amino acid sequences (LSaa) and nucleotide sequences (LSnt). Four datasets of partial sequences (90 to 60% of full length) were generated for both amino acid and nucleotide sequences to test the flexibility of the model for variable sequence/contig lengths (“Methods”). This resulted in five LSaa and five LSnt datasets.

**Fig. 5:**
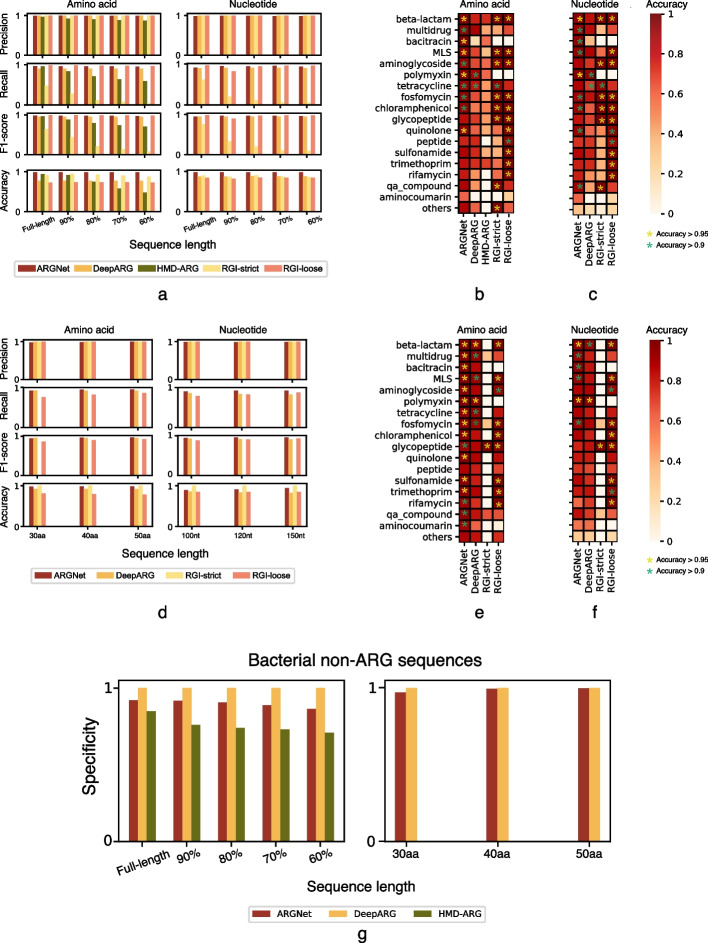
Performance of the deep learning models on test datasets. Precision, recall, F1 score, and classification accuracy of long sequences **a** and short sequences **d**. Left, amino acids; right, nucleotides. Datasets are indicated as proportion of the full-length sequences **a** and sequence length **d**. Classification accuracy of long amino acid sequences **b**, long nucleotide sequences **c**, short amino acid sequences **e**, and short nucleotide sequences **f** by antibiotic category. Nineteen minor ARG categories (with fewer than 50 sequences) are combined and displayed as one category called “others.” Accuracy level is indicated by asterisk with different colors. Identification specificity of bacterial non-ARG amino acid sequences **g**. Left, long sequence; right, short sequence. Datasets are indicated as proportion and sequence length, respectively

ARGNet had average precision, recall, and F1 values (“Methods”) of 0.994, 0.960, and 0.977 (with minima of 0.989, 0.952, and 0.970) over the five LSaa datasets (Fig. 5a). Consistent results were observed across the datasets of different lengths. DeepARG had higher average precision (1.000) but lower recall (0.902) and F1 (0.949) scores. HMD-ARG demonstrated relatively inferior performance, indicated by lower average precision, recall, and F1 scores (0.911, 0.747, and 0.817). Its performance was affected by the completeness of the input sequences. A comparison was also conducted with the best hit approach, where the Resistance Gene Identifier (RGI) was evaluated. RGI attained an average prefect precision (1.000) but exhibited low recall (0.197) and F1 score (0.301) with using “prefect and strict hits only” criteria. When employing the criteria of “perfect, strict, and loose hits,” RGI achieved perfect precision (1.000) along with high recall (0.979) and F1 score (0.989). According to the five LSnt datasets, a similar pattern of performance was observed. Average precision, recall, and F1 score were found to be 0.989, 0.938, and 0.963, respectively, for ARGNet, while 1, 0.900, and 0.947, respectively, for DeepARG (Fig. 5a). Performance of HMD-ARG on ARG prediction using nucleotide sequences was not computed as it did not accept nucleotide sequences as input. In the “perfect and strict hits only” criteria, RGI achieved precision, recall, and F1 score values of 1.000, 0.208, and 0.302, respectively. When considering the criteria of “perfect, strict, and loose hits,” RGI demonstrated precision, recall, and F1 score values of 1.000, 0.927, and 0.961, respectively. ARGNet outperformed DeepARG, HMD-ARG, and RGI (under both strict and loose criteria) at classification on the LSaa dataset where average accuracies were 0.986, 0.770, 0.733, 0.915 (strict), and 0.733 (loose), respectively, and it outperformed DeepARG and RGI (under both strict and loose criteria) on the LSnt dataset (0.976 vs 0.878 vs 0.879 (strict) vs 0.836 (loose)) (Fig. 5a).

Based on five LSaa datasets, ARGNet exhibited higher accuracy than the other two deep learning models for all categories (Fig. 5b). Consistent results were observed across the truncated sequence sets. An average classification accuracy > 0.9 was achieved in 10 categories with polymyxin (0.995), bacitracin (0.993), beta-lactam (0.981), quinolone (0.959), and MLS (0.958) having average accuracy values > 0.95. DeepARG achieved high average classification accuracy (> 0.9) in only two categories. While HMD-ARG predicted eight categories with accuracy > 0.9 in the full-length dataset, it performed poorly on the truncated sequences for all categories (average accuracies were 0.56, 0.35, 0.21, and 0.14 for 90%, 80%, 70%, and 60% of full-length dataset, respectively). In terms of loose criteria, RGI exhibited an additional category with an accuracy greater than 0.9 in comparison to ARGNet. However, under strict criteria, RGI displayed one fewer category with an accuracy exceeding 0.9 when compared to ARGNet.

For the five LSnt datasets, ARGNet showed high classification performance (average accuracy > 0.9) in nine categories, while DeepARG achieved high accuracy in only two categories. The top five predictions in ARGNet were polymyxin (0.981), beta-lactam (0.965), bacitracin (0.948), MLS (0.943), and quinolone (0.931) (Fig. 5c). ARGNet outperformed DeepARG in 17 of the 18 antibiotic resistance categories. ARGNet had two fewer categories with accuracy > 0.9 than RGI in the loose criteria and had the same number of categories with accuracy > 0.9 with RGI in the strict criteria (Fig. 5c).

### Prediction of short reads

ARGNet-S was designed to predict ARGs from short sequence (SS; sequences with 30–50 amino acids or 100–150 nucleotides). The short-read test datasets (SSaa and SSnt; “Methods”) were generated from ARG-test-db and negative sequence datasets (of both amino acid and nucleotide sequences) in short-sequence format. There were three SSaa datasets with sequence lengths of 30, 40, and 50 amino acids and three SSnt datasets with lengths of 100, 120, and 150 nucleotides. Performance of HMD-ARG on short sequence prediction (both amino acid and nucleotide datasets) was not computed as it did not accept short sequences.

Over the three SSaa datasets, ARGNet achieved average precision, recall, and F1 score of 0.986, 0.936, and 0.960, respectively. As with SSnt datasets, these values were 0.989, 0.919, and 0.952, respectively. DeepARG obtained average precision, recall, and F1 scores of 1, 0.920, and 0.958 on SSaa and 1, 0.842, and 0.914 on SSnt. RGI attained an average precision, recall, and F1 score of 1.000, 0.004, and 0.008, respectively, under the strict criteria and 1.000, 0.812, and 0.896, respectively, under the loose criteria on SSaa. On SSnt, RGI achieved an overall precision, recall, and F1 score of 1.000, 0.003, and 0.007, respectively, in the strict criteria and 1.000, 0.834, and 0.909, respectively, in the loose criteria (Fig. 5d).

The accuracy of ARGNet was higher than that of DeepARG and RGI under the loose criteria, across the different lengths of both SSaa and SSnt (Fig. 5d) with average accuracies of ARGNet vs DeepARG vs RGI (loose) of 0.980 vs 0.918 vs 0.795 for SSaa and 0.911 vs 0.838 vs 0.848 for SSnt. RGI, when evaluated using the strict criteria, demonstrated a higher classification accuracy primarily due to its identification of a limited number of ARG categories, encompassing only a small number of sequences, for both the SSaa and SSnt datasets.

On the three SSaa datasets, ARGNet had higher average accuracies than deepARG and RGI under both strict and loose criteria, in all ARG categories (Fig. 5e). ARGNet obtained an average accuracy > 0.9 in 16 categories, while DeepARG achieved such average accuracy in 6 categories. In ARGNet, the highest accuracy was observed in polymyxin (0.995), beta-lactam (0.995), and bacitracin (0.994), while multidrug, MLS, chloramphenicol, quinolone, sulfonamide, aminoglycoside, fosfomycin, tetracycline, trimethoprim, and qa_compound also demonstrated accuracy > 0.95. Under the loose criteria, RGI demonstrated nine categories with a prediction accuracy greater than 0.9. However, under the strict criteria, RGI had only one category with an accuracy exceeding 0.9. Within the three SSnt datasets, ARGNet outperformed DeepARG (Fig. 5f) with 6 well-classified categories (accuracy > 0.9). They were polymyxin (0.98), beta-lactam (0.952), bacitracin (0.934), MLS (0.929), multidrug (0.921), and fosfomycin (0.911). Under the loose criteria, RGI exhibited 9 categories with a prediction accuracy greater than 0.9. However, under the strict criteria, RGI had only one category with an accuracy exceeding 0.9.

### Validation through bacterial non-ARG sequence dataset

Virus sequences were used as a “non-ARG” dataset (negative samples) to determine the cutoff and evaluate ARG identification in the autoencoder model. The specificity of ARGNet to distinguish non-ARG sequences was further evaluated using bacterial non-ARG (amino acid) sequence dataset as they were likely to have more intrinsic affinity to the bacterial ARG sequences. ARGNet, DeepARG, and HMD-ARG had average identification specificity of 0.900, 1.000, and 0.758, respectively, for the 5 LSaa datasets and 0.989 and 0.999 (ARGNet and DeepARG) on the 3 SSaa datasets (Fig. 5g). DeepARG achieved such high specificity because there is no well-defined non-ARG database, and the test dataset here was built from sequences with no BLAST hits to ARGNet-DB (“Methods”).

### Prediction of ESKAPE sequences

To evaluate the performance of ARGNet on ESKAPE sequences with varying sequence identities to ARGNet-DB, DIAMOND analysis was conducted. The analysis involved using ESKAPE sequences as queries and ARGNet-DB as the reference. The sequence identities were categorized into four ranges: (0, 30), [30, 40), [40, 50), and [50, 60), representing less contrast and intermediate hits. The test result is presented in Table 1. The result indicates that sequences falling within higher identity ranges have a greater likelihood of being predicted as ARGs. In the identity range of (0, 30), only 0.41% and 0.09% (considering the classification probability equal to or greater than 0.8) of sequences were predicted as ARGs, suggesting that ARGNet may not exhibit excessive sensitivity during identification.

**Table 1:** Result of ARGNet test on ESKAPE sequences

Identity range	Prediction of ARG (%)	Classification of ARG with probability > = 0.8 (%)
(0, 30)	0.41	0.09
[30, 40)	1.35	0.52
[40, 50)	6.20	3.38
[50, 60)	14.27	9.14

### Prediction of *E. coli* K12 sequences

ARGNet predicted that 9.15% (5.04% when considering only predictions with a probability greater than or equal to 0.8) of all the *E. coli* K12 testing sequences were ARGs. The RGI program on the same dataset predicted 1.61% of the test sequences as “Perfect and Strict hits only” ARGs and 8.00% as “Perfect, Strict, and Loose hits” ARGs. The classification of ARGs into different categories is visually represented in Fig. S9a. Among the 5349 protein sequences in the dataset, a total of 393 sequences were annotated as “transporter.” Notably, 5.85% of these transporter sequences were predicted as ARGs by ARGNet (5.60% when considering only predictions with a probability greater than or equal to 0.8), whereas RGI predicted 25.95% and 3.05% of the transporter sequences as ARGs under the “Perfect, Strict, and Loose hits” and “Perfect and Strict hits only” modes, respectively. The classification of ARGs within the transporter subset is visually depicted in Fig. S9b.

### Prediction of phylogenetically inferred *mcr* genes

**Fig. 6:**
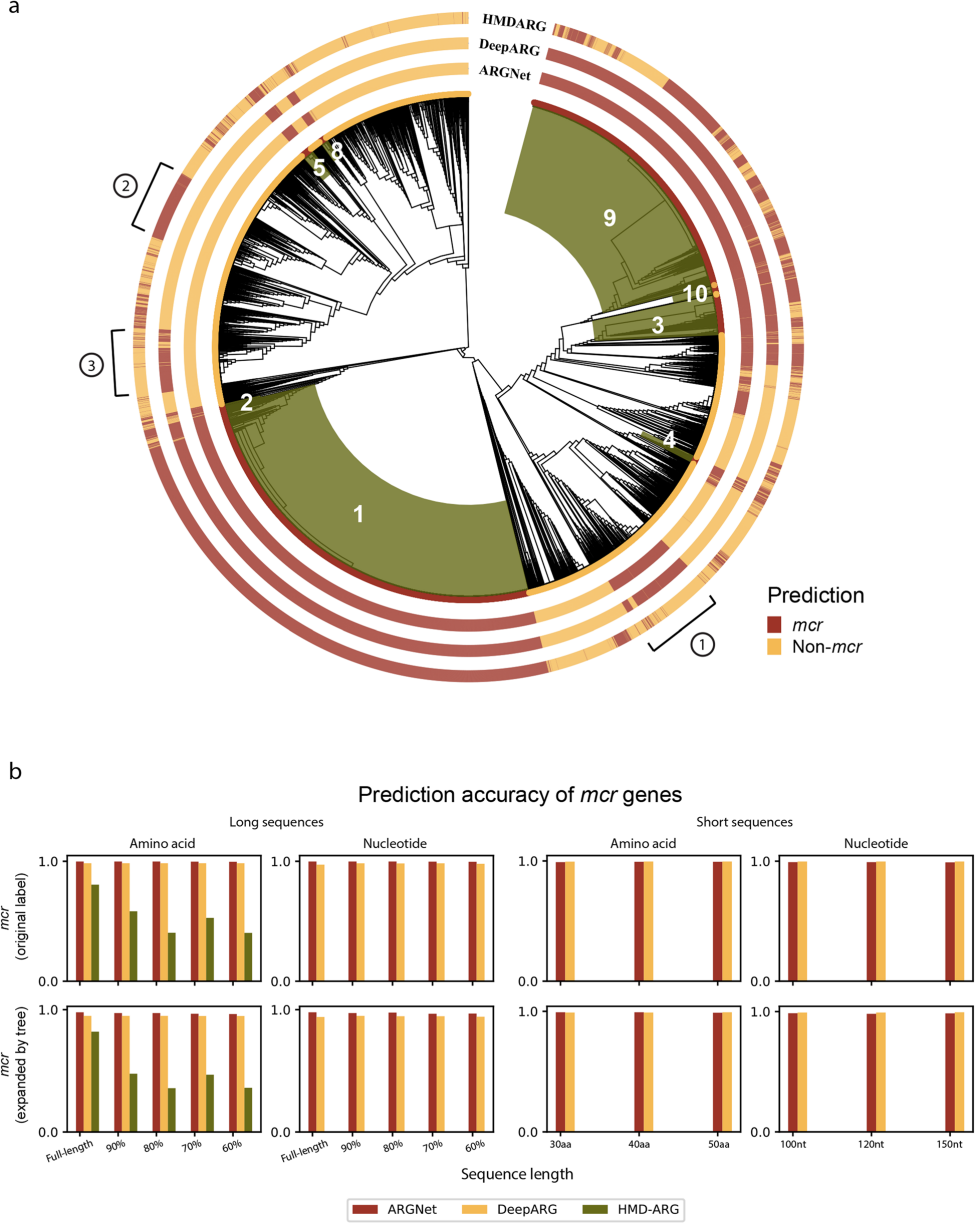
Prediction of *mcr* genes. **a** Phylogenetic tree of *mcr*-like dataset. Tree designation (inner circle) and deep learning model predictions (outer circles) are annotated for each sequence, with different colors (red for *mcr*, yellow for non-*mcr*). The clade of each subtype of *mcr* genes is shaded in green and labelled in white. The three major groups of inconsistent prediction across models are marked with circled number. **b** Prediction accuracy of *mcr* genes on long amino acid and nucleotide sequences (left) and short amino acid and nucleotide sequences (right). Accuracy was calculated and displayed for *mcr*-labelled genes and expanded *mcr* genes, respectively

Based on curation of ARGs mostly from cultured pathogens, the current antibiotic resistance databases (e.g., CARD) provide standard collection of resistance determinants, while they may not accommodate the fast-evolving resistome from metagenomic datasets. Phylogenetic approach was applied to expand the definition of the rapidly disseminated mobilized colistin resistance genes (*mcr*, *mcr*-1 to *mcr*-10). The *mcr* genes have been actively researched in the recent years, and some newly identified *mcr* genes were reported in the literature but not yet documented in the databases, e.g., *mcr-9* and *mcr-10* were reported in 2022, but they were not yet in the CARD database at the time when the authors conducted this research. A phylogenetic tree was constructed (Fig. 6a, “Methods”) for 17,200 *mcr*-like sequences, from which 8403 sequences (7865 originally labelled as *mcr* genes and 538 expanded by tree) were designated as *mcr* genes based on their phylogenetic placement (under the same clades with known *mcr* genes; “Methods”). Long and short *mcr*/*mcr-like* amino acid and nucleotide datasets (with variation in sequence length) were generated using the same approach as in generating the LSaa/LSnt/SSaa/SSnt datasets. Based on phylogenetic designation, the prediction accuracy was compared among three deep learning models.

Based on the long sequence amino acid datasets, ARGNet, DeepARG, and HMD-ARG had average prediction accuracies of 0.997, 0.990, and 0.545, respectively, for the 7865 *mcr*-labelled sequences and 0.970, 0.950, and 0.497, respectively, for the 538 expanded *mcr* sequences. For the long nucleotide sequences, ARGNet and DeepARG had accuracies of 0.997 and 0.980 and 0.974 and 0.945 for these two sets of *mcr* sequences, respectively (Fig. 6b).

For the short sequence datasets, average accuracies for ARGNet and DeepARG were 0.996 and 0.998 for amino acids and 0.991 and 0.997 for nucleotides on the *mcr*-labelled genes. As with the expanded *mcr* genes, the accuracies for ARGNet and DeepARG were 0.998 and 0.996 for amino acids and 0.984 and 0.993 for nucleotides (Fig. 6b). ARGNet achieved good consistency of prediction accuracy (> 0.95) across all the *mcr* test datasets.

Although not annotated as *mcr* based on phylogenetic tree, three groups of sequences were inconsistently predicted as *mcr* by either one or two of the testing deep learning models (Fig. 6a). Further experimental validation will indicate the potential of deep learning models to identify undetermined ARGs.

### Potential of novel ARG detection

**Fig. 7:**
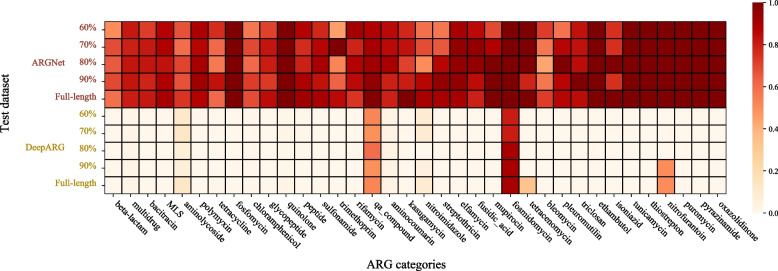
Sensitivity of quasi-negative test. The quasi-negative test sensitivity of ARGNet (upper part, separated by datasets of different sequence length) and DeepARG (lower part, separated by datasets of different sequence length) for each ARG category was shown in heatmap

A quasi-negative test (“Methods”) was conducted on long amino acid dataset from each category of ARGs. The result is presented in Fig. 7. The performance of ARGNet is better than DeepARG in all 36 ARG categories. The average sensitivity was higher than 0.6 for ARGNet across all ARG categories. There were 14 categories possessing average sensitivity higher than 0.9. They were fosfomycin, quinolone, ethambutol, tunicamycin, nitrofurantoin, puromycin, thiostrepton, pyrazinamide, oxazolidinone, fosmidomycin, tetracenomycin, elfamycin, qa_compound, and mupirocin, with the first 10 categories fully identified as ARG in the test. There were two categories (fosmidomycin and qa_compound) with average sensitivity higher than 0.5 in DeepARG.

### Runtimes

The wall time of the prediction process between ARGNet and DeepARG was compared. ARGNet and DeepARG were run on the same machine and utilized 48 Intel(R) Xeon(R) Gold 6252 CPU cores and one Tesla V100 GPU card when program running. Four representative LSaa/LSnt/SSaa/SSnt datasets were used. ARGNet outperformed DeepARG in all tests, with 39.5–57.0% shorter runtime across the four datasets (Table 2).

**Table 2:** Runtime test of ARGNet and DeepARG

Test dataset (sequence type | long or short | sequence number | dataset size)	Runtime (mm:ss)		Time saved (%)
	ARGNet	DeepARG	
Amino acid | full length | 5690 | 2.3 M	00:42	01:32	54.0
Nucleotide | full length | 3567 | 4.3 M	00:28	00:50	44.0
Amino acid | short reads | 82,350 | 7.5 M	01:01	02:22	57.0
Nucleotide | short reads | 53,505 | 9.4 M	00:55	01:31	39.5

ARGNet and DeepARG were evaluated on four datasets, long or short amino acid, or nucleotide sequences. The number of sequences and size of the data set (megabytes) are indicated. Relative time saved by ARGNet was calculated as the difference between the runtimes of DeepARG and ARGNet, divided by the runtime of DeepARG.

## Discussion and conclusions

A neural network that identifies and classifies antibiotic resistance genes from both short reads and contigs of different lengths has been developed. ARGNet does not require alignments and outperforms other deep learning methods (DeepARG and HMD-ARG) and best hit approach in most tests.

The pursuit of the optimal approach to classify biological sequences is always addressing the balance between false positive and negative. Alignment-based approach (e.g., BLAST) has been widely used for ARG identification and classification based on the identity of nucleotide/amino acid sequences against reference database [13, 39]. In practice, an identity cutoff was normally set to 80% as it greatly minimizes the false-positive rate [40]. By adopting the similarity distribution from BLAST into deep learning framework, DeepARG clearly filtered out non-ARG sequences, resulting in perfect precision in different test datasets in this study. However, it still inherits the disadvantage of alignment-based approach and attenuate the robustness to detect novel ARGs [13]. In this study, by adopting autoencoder model, ARGNet showed comparable precision and superiority in both recall and F1 score in the identification of ARGs. It also demonstrated great potential to recognize novel ARGs, justified by the detection of quasi-negative ARGs (see “Results,” Fig. 7). Autoencoders have been widely used to learn representations of sets of data, especially where a reduction in dimensionality is needed, by using a trained network to remove noise and compress information (e.g., for face recognition [41] and anomaly detection [42, 43]). The autoencoder model captures representative features from the reference ARG sequences through multiple layers of dimension reduction (encoding, Additional file 1: Fig. S9), which can be expanded to identify distantly related ARGs. A potential caveat is that the identification threshold of ARGNet was determined based on only the ARGNet-DB and curated virus sequences, which may introduce bias to differentiate other non-ARG sequences. Nonetheless, using the same threshold, ARGNet was also able to recognize non-ARG bacterial sequences (see “Results,” Fig. 5g) which demonstrated the robustness and effectiveness of the proposed model. The robustness of the autoencoder model in identifying ARGs suggests that deep learning model may be useful in sequence searching comparable to the state-of-the-art approaches.

Compared to other two models, ARGNet showed generally higher accuracy in ARG classification across different test datasets (LSaa/LSnt/SSaa/SSnt). In principle, these three tools used three different model architectures. While ARGNet used a one-dimensional CNN model structure, DeepARG used a dense network, and HMD-ARG used a two-dimensional CNN model structure. CNNs have been widely used to characterize and classify raw sequence data [44] as they outperform traditional machine learning methods by recognizing features directly from raw sequences, avoiding human-defined feature generation. The outperformance of ARGNet against the other two tools may indicate the superiority of using one-dimensional CNN in “one-dimensional” biological sequences such as nucleotide and protein, which has been well-known for sequence motif detection (Additional file 1: Fig. S10, Table S5) and used in classification of different biological sequences [45, 46]. The classification accuracy was slightly decreased in ARG categories with fewer sequences, which may be due to the issues from the multiclass imbalanced data [47, 48]. There were 13 categories with < 10 sequences available in the current database. With the continuous efforts on sequencing and antibiotics resistance test, more entries will be included to enrich the training database, which should result in an improved classification performance.

With the development and advance of NGS technology, shotgun metagenomic sequencing has been used in investing emergence of infectious disease including viruses and antibiotic-resistant bacteria [39, 46]. Due to the limited read length of NGS, metagenomic sequencing reads are not able to generate complete gene sequences of interest, in the case of AMR or ARGs. Consequently, two strategies have been used for ARG identification, based on short reads or assembled contigs [49]. Comparatively, the latter strategy is thought to be more accurate as the longer assembled contigs contain more information. In this study, ARGNet-S and ARGNet-L were implemented for both strategies. According to the performance test, ARGNet performed consistently well across different datasets (both assembled contigs with different completeness and short reads of different sequence lengths), supporting its usage in different application scenarios, even without the requirement of the time-consuming sequence assembly [50].

Antibiotic-resistant bacteria becomes ubiquitous in the world, leading to a public health threat of drug-resistant infections. Multidrug resistance was identified in various clinical bacteria which may result in increased mortality and also cost in treatment [51]. Without the appropriate diagnosis of the ARG type, it is likely to lead to limited antibiotic treatments and delay the control of pathogens. Hence, it is essential to develop rapid ARG detection methods for both clinical and environmental settings [52]. In this study, ARGNet demonstrated superior time efficacy. ARGNet utilized batch processing which operates sequences with different batch sizes at one time for speeding up prediction. In the era of big data, it is common to receive large volume of sequences. ARGNet can take use of computational resources (such as GPU memory). The application of ARGNet is efficient and scalable, without pre-processing steps such as sequence assembly (for ARGNet-S) and BLAST alignment.

ARGNet was developed to identify ARG genes and further classify into 36 categories based on nucleotide and amino acid sequence. It was not designed to predict antibiotic resistance derived from SNPs. Although ARGNet demonstrated capability to detect distantly related (novel) ARGs (quasi-negative test), it cannot explicitly classify ARGs from entirely novel category that out of the current classification scheme. As with other deep learning models, the performance of ARGNet is relied on the quality of database, which is hinged by the intrinsic characteristics (e.g., lack of negative dataset, imbalanced data of different ARG categories) of the database. Currently, ARGs are identified mainly based on alignment-based methods, with the implicit assumption that ARGs are determined by the sequence similarity against reference database. However, for ARGs that function as proteins, their three-dimensional structural form will be important in determining their resistance activity [53]. Prediction based on three-dimensional structure has identified thousands of ARGs distantly related to the known ones [54]. As an alignment-free deep learning model, ARGNet provides a framework compatible with functional annotation derived from structure-based evidence. It can provide in silico prediction of ARGs that may be distant in sequence from those in existing ARG databases. These can then be used to search for potential novel ARG candidates in sequences from real samples (such as humans, animals, or the environment), which could be validated via experiments.

In addition, ARGNet provides a promising and efficient approach for ARG identification and classification in multiple application scenarios, which can be further extended. For example, third-generation sequencing technology such as nanopore sequencing has been widely used for ARG discovery [55]. Although possessing high error rate, it is reputed by the fast turnaround time, real-time sequencing, and portability [56]. The extension of our deep learning model into error-corrected long-read prediction of ARGs will provide a powerful tool for timely monitoring of antibiotic-resistant bacteria.

Genetic sequence identification and classification approaches offer a number of advantages over the classical culture-based AST method. However, current sequence-based approaches are also limited by the inability to link the ARGs, with absolute confidence, to their hosting bacterial genomes and cells. Long-read sequencing technologies, such as nanopore sequencing, can be used to identify the bacterial genomes and species in which chromosomal ARGs are located. For ARGs located in plasmids, single-cell deep sequencing can reveal their association. However, it is important to note that the presence of ARGs may not be the sole determinant of resistant phenotypes. Therefore, interpreting the clinical implications based on the ARGs identified from metagenomic data requires caution.

### Supplementary Information

**Additional file 1: Fig. S1.** Phylogenetic tree of mcr-1 gene. **Fig. S2.** Phylogenetic tree of mcr-2 gene. **Fig. S3.** Phylogenetic tree of mcr-3 gene. **Fig. S4.** Phylogenetic tree of mcr-4 gene. **Fig. S5.** Phylogenetic tree of mcr-5 gene. **Fig. S6.** Phylogenetic tree of mcr-8 gene. **Fig. S7.** Phylogenetic tree of mcr-9 gene. **Table S1.** Autoencoeder in ARGNet-L. Adam optimizer was utilized with learning rate 1e-4 and trained with batch-size of 256. **Table S2.** Convolutional neural network (CNN) in ARGNet-L. Adam optimizer was utilized with learning rate of 0.001 and trained with batch-size of 256. **Table S3.** Autoencoeder in ARGNet-S. Adam optimizer was utilized with learning rate of 1e-4 and trained with batch-size of 2048. **Table S4.** CNN in ARGNet-S. Adam optimizer was utilized with learning rate 0.001 (learning rate decay of 0.001) and trained with batch-size of 2048. **Table S5.** Weight sum distribution along sequence position of ARGs. **Fig. S8.** Phylogenetic tree of mcr-10 gene. **Fig. S9.** Prediction on *E. coli* K12 sequences with ARGNet and RGI. a Classification results of the *E. coli* K12 sequences identified as ARGs by ARGNet and RGI. b Classification results of the transporters sequences from *E. coli* K12 identified as ARGs by ARGNet and RGI. **Fig. S10.** Latent space representation of autoencoder. The upper part is the illustration of autoencoder model. W denotes the weight of each neuron learned and optimized from the input data. The lower part is dimension reduction via tSNE of the latent space extracted from the ARGNet’s autoencoder trained by our ARG reference data (*n*=21971). **Fig. S11.** The weight sum distribution along sequence position in the latent space. Each boxplot represents the weight sum distribution of all test sequences in each category. The red line chart shows the number of sequences (right y-axis) on each range of positions (x-axis). The highest weight sum position ranges (x-axis) supported by enough number (> 90%) of sequences are highlighted in orange.

## Data Availability

ARGNet consists of a command line program where the input is in the format of FASTA. The program, models, and test data are on https://github.com/id-bioinfo/ARGNet. The online service of ARGNet could be found on https://argnet.hku.hk/.
