# Supplementary material for: ARGNet: using deep neural networks for robust identification and classification of antibiotic resistance genes from sequences
Source: Microbiome. 2024 May 9;12:84. doi: 10.1186/s40168-024-01805-0 (PMC11080312; doi:10.1186/s40168-024-01805-0)
Supplement: Supplementary file 2 — Additional file 1: Fig. S1. Phylogenetic tree of mcr-1 gene. Fig. S2. Phylogenetic tree of mcr-2 gene. Fig. S3. Phylogenetic tree of mcr-3 gene. Fig. S4. Phylogenetic tree of mcr-4 gene. Fig. S5. Phylogenetic tree of mcr-5 gene. Fig. S6. Phylogenetic tree of mcr-8 gene. Fig. S7. Phylogenetic tree of mcr-9 gene. Table S1. Autoencoeder in ARGNet-L. Adam optimizer was utilized with learning rate 1e-4 and trained with batch-size of 256. Table S2. Convolutional neural network (CNN) in ARGNet-L. Adam optimizer was utilized with learning rate of 0.001 and trained with batch-size of 256. Table S3. Autoencoeder in ARGNet-S. Adam optimizer was utilized with learning rate of 1e-4 and trained with batch-size of 2048. Table S4. CNN in ARGNet-S. Adam optimizer was utilized with learning rate 0.001 (learning rate decay of 0.001) and trained with batch-size of 2048. Table S5. Weight sum distribution along sequence position of ARGs. Fig. S8. Phylogenetic tree of mcr-10 gene. Fig. S9. Prediction on E. coli K12 sequences with ARGNet and RGI. a Classification results of the E. coli K12 sequences identified as ARGs by ARGNet and RGI. b Classification results of the transporters sequences from E. coli K12 identified as ARGs by ARGNet and RGI. Fig. S10. Latent space representation of autoencoder. The upper part is the illustration of autoencoder model. W denotes the weight of each neuron learned and optimized from the input data. The lower part is dimension reduction via tSNE of the latent space extracted from the ARGNet’s autoencoder trained by our ARG reference data (n=21971). Fig. S11. The weight sum distribution along sequence position in the latent space. Each boxplot represents the weight sum distribution of all test sequences in each category. The red line chart shows the number of sequences (right y-axis) on each range of positions (x-axis). The highest weight sum position ranges (x-axis) supported by enough number (> 90%) of sequences are highlighted in orange. [file 40168_2024_1805_MOESM1_ESM.pdf]

Residual block

The layers in ResBlock were implemented as:
" BatchNormalization - RELU - Conv1d - l2 regularizers - BatchNormalization - RELU - Conv1D ".

Supplementary Tables

Table S1 Autoencoeder in ARGNet-L. Adam optimizer was utilized with learning rate 1e-4 and trained with batch-size of 256.

Model	Layer	Kernel number	Kernel size
Encoder	Conv1D	512	20
	BatchNormalization	NA	NA
	RELU	NA	NA
	ResBlock	512, 128	9
	MaxPooling	NA	2
	ResBlock	512, 128	9
	MaxPooling	NA	2
	Conv1D	256	8
	BatchNormalization	NA	NA
	RELU	NA	NA
	ResBlock	256, 64	8
	MaxPooling	NA	2
	ResBlock	256, 64	8
	MaxPooling	NA	2
	ResBlock	256, 64	7
	MaxPooling	NA	2
	ResBlock	265, 64	7
	MaxPooling	NA	2
Decoder	ResBlock	256, 64	7
	Upsampling	NA	2
	ResBlock	256, 64	7
	Upsampling	NA	2
	ResBlock	256, 64	8
	Upsampling	NA	2
	ResBlock	256, 64	8
	Upsampling	NA	2
	Conv1D	512	20
	BatchNormalization	NA	NA
	ResBlock	512, 128	9
	Upsampling	NA	2
	ResBlock	512, 128	9
	Upsampling	NA	2
	Conv1D	512	20
	Conv1D	22	12

Table S2 Convolutional neural network (CNN) in ARGNet-L. Adam optimizer was utilized with learning rate of 0.001 and trained with batch-size of 256.

Model	Layer	Kernel number	Kernel size
CNN	Conv1d	512	20
	Maxpooling	NA	4
	Conv1d	512	15
	Maxpooling	NA	2
	Conv1d	512	12
	Conv1d	512	12
	GlobalAveragePooling	NA	NA
	Dense	512	NA
	Dense	36	NA

Table S3 Autoencoeder in ARGNet-S. Adam optimizer was utilized with learning rate of 1e-4 and trained with batch-size of 2048.

Model	Layer	Kernel number	Kernel size
Encoder	Conv1D	400	8
	BatchNormalization	NA	NA
	RELU	NA	NA
	ResBlock	400, 400	4
	ResBlock	400, 400	4
	MaxPooling	NA	2
	Conv1D	200	3
	BatchNormalization	NA	NA
	RELU	NA	NA
	ResBlock	200, 200	3
	ResBlock	200, 200	3
	MaxPooling	NA	2
	ResBlock	200, 200	3
	MaxPooling	NA	2
	ResBlock	200, 200	3
	MaxPooling	NA	2
Decoder	ResBlock	200, 200	3
	Upsampling	NA	2
	ResBlock	200, 200	3
	Upsampling	NA	2
	ResBlock	200, 200	3
	Upsampling	NA	2
	ResBlock	200, 200	3
	Conv1D	400	8
	BatchNormalization	NA	NA
	RELU	NA	NA
	ResBlock	400, 400	4
	Upsampling	NA	2
	ResBlock	400, 400	4
	ResBlock	400, 400	4
	ResBlock	400, 400	4
	ResBlock	400, 400	4

	Conv1D	400	8
	Conv1D	22	5

Table S4 CNN in ARGNet-S. Adam optimizer was utilized with learning rate 0.001 (learning rate decay of 0.001) and trained with batch-size of 2048.

Model	Layer	Kernel number	Kernel size
CNN	SeparableConv1D	512	25
	BatchNormalization	NA	NA
	SeparableConv1D	512	20
	BatchNormalization	NA	NA
	SeparableConv1D	512	15
	BatchNormalization	NA	NA
	SeparableConv1D	512	12
	SeparableConv1D	512	12
	GlobalAveragePooling	NA	NA
	Dense	512	NA
	GlobalAveragePooling	NA	NA
	Dense	256	NA
	GlobalAveragePooling	NA	NA
	Dense	128	NA
	GlobalAveragePooling	NA	NA
	Dense	64	NA
	Dense	36	NA

Table S5 Weight sum distribution along sequence position of ARGs.

ARGs category	Position	Weight sum (median)	Number of sequences	Sequence length (median)	Position proportion*
**Beta-lactam**	161-192	464.94	1732	291	0.55
**Multidrug**	225-256	472.44	987	413	0.54
**Bacitracin**	161-192	573.13	852	275	0.59
**MLS**	193-224	474.11	507	498	0.39
**Aminoglycoside**	161-192	437.42	332	259	0.62
**Polymyxin**	193-224	477.99	207	660	0.29
**Tetracycline**	193-224	478.66	130	388	0.50
**Fosfomycin**	65-96	426.08	129	140	0.46
**Chloramphenicol**	97-128	469.20	112	216	0.45
**Glycopeptide**	161-192	529.03	96	294	0.55
**Quinolone**	129-160	480.02	78	226	0.57
**Peptide**	257-288	535.36	45	541	0.48
**Sulfonamide**	161-192	479.53	35	279	0.58
**Trimethoprim**	33-64	396.01	36	157	0.21

*Position proportion is calculated as start position divided by median sequence length, for example, the position proportion of beta-lactam is 161/291.

For class B betalactamase, Timothy Palzkill, et al. (2012) found that 120th-263th amino acids constructed the active site.

Supplementary Figures

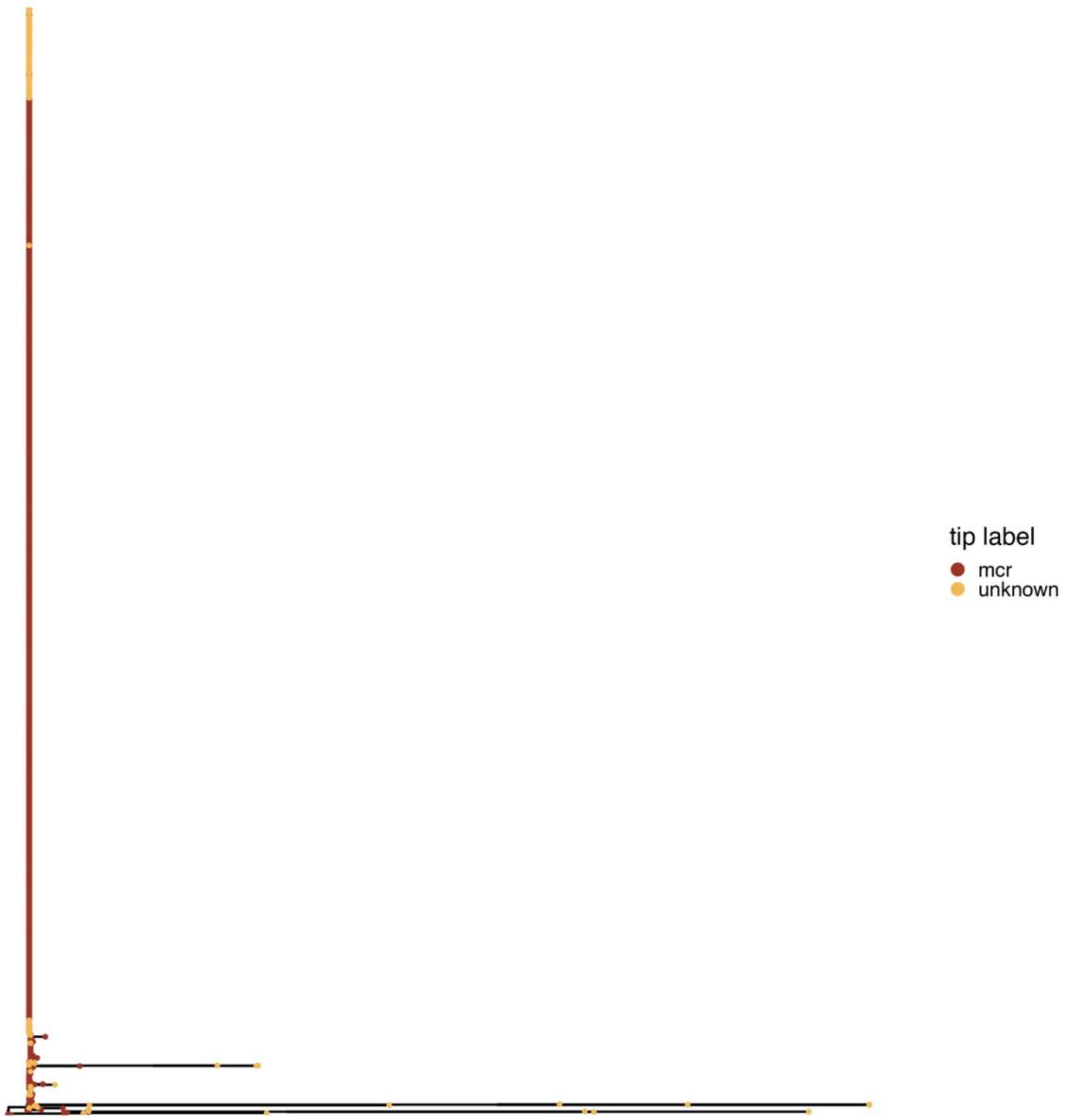

Fig. S1 Phylogenetic tree of *mcr-1* gene.

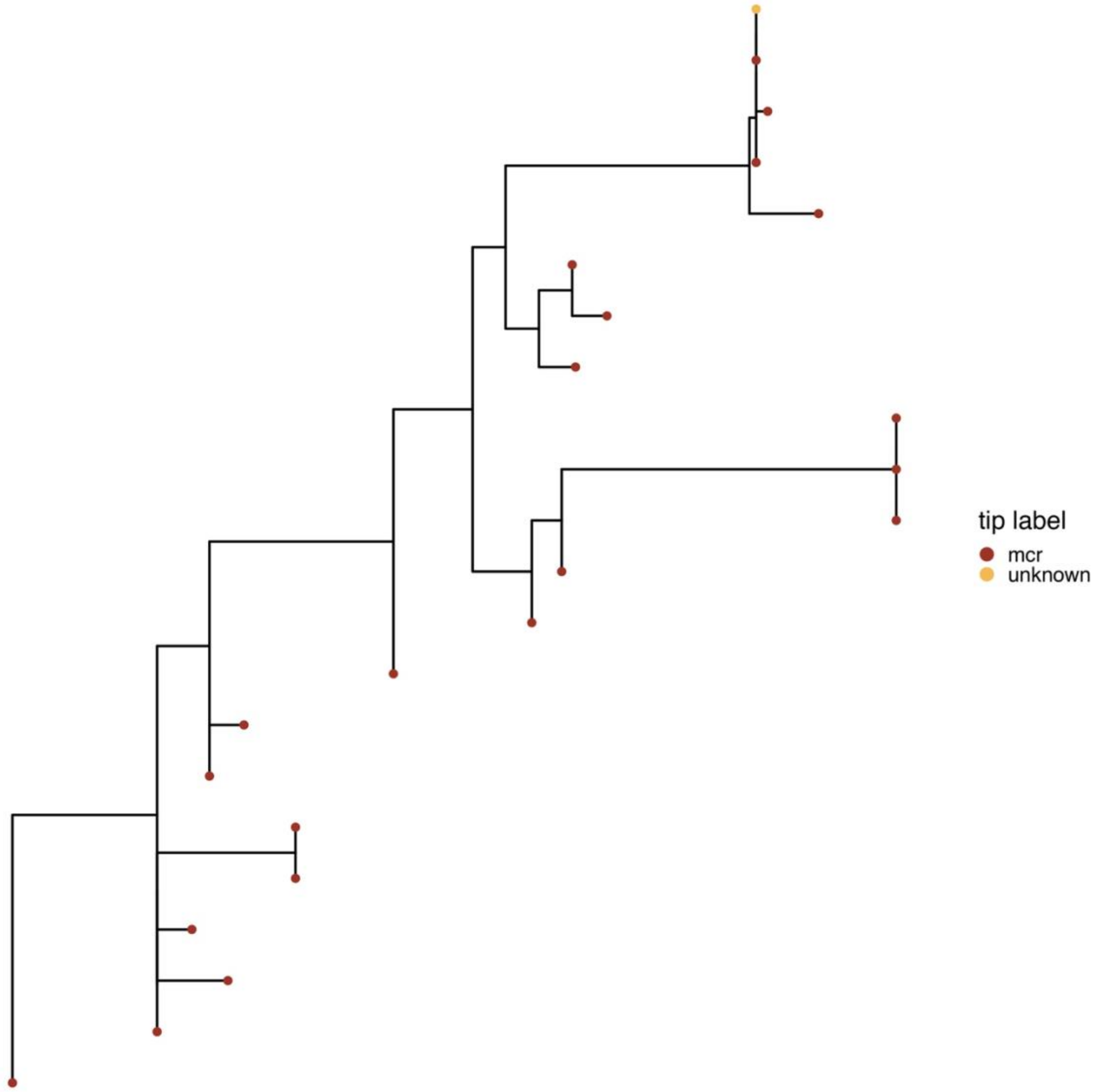

Fig. S2 Phylogenetic tree of *mcr-2* gene.

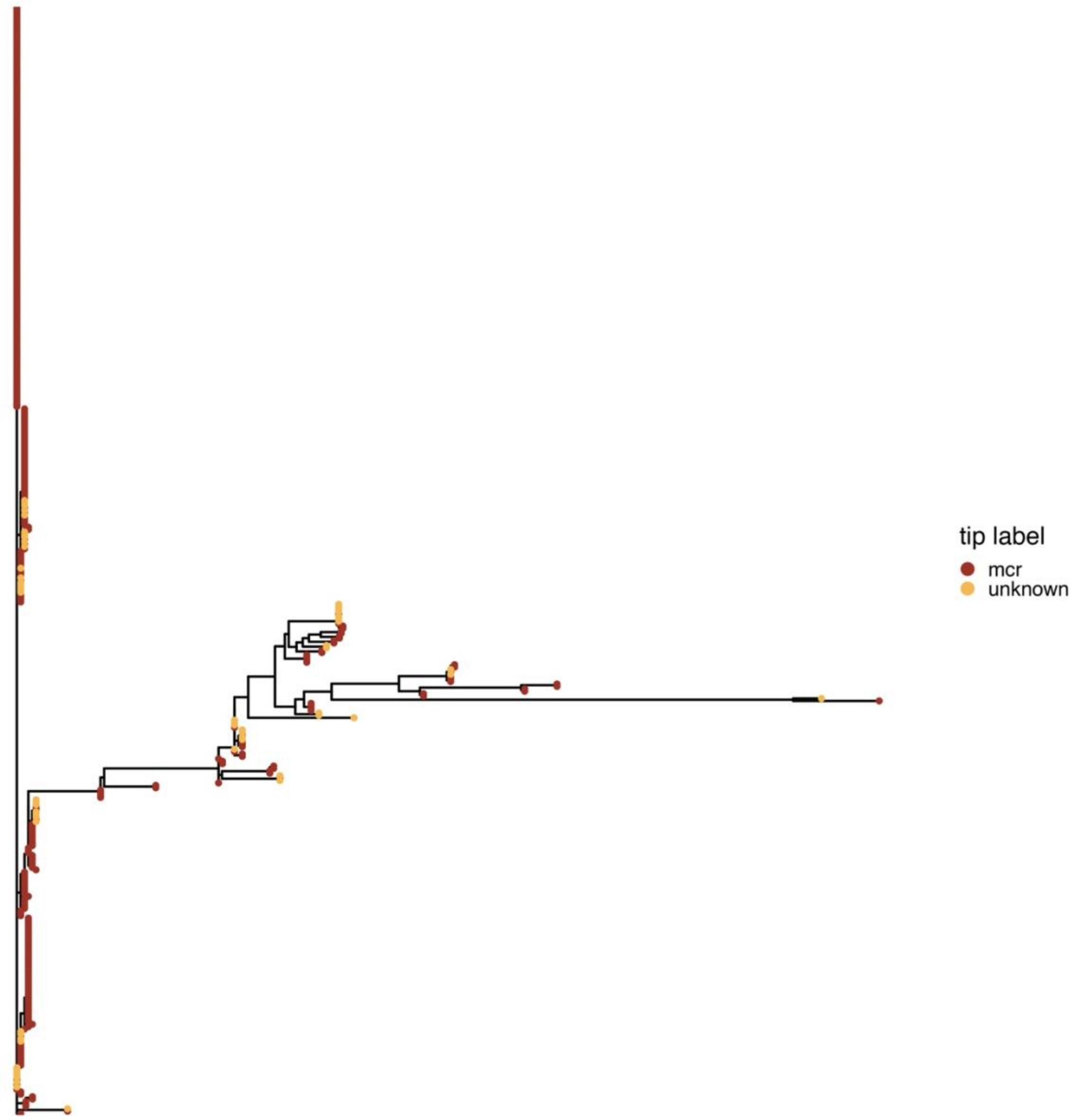

Fig. S3 Phylogenetic tree of *mcr-3* gene.

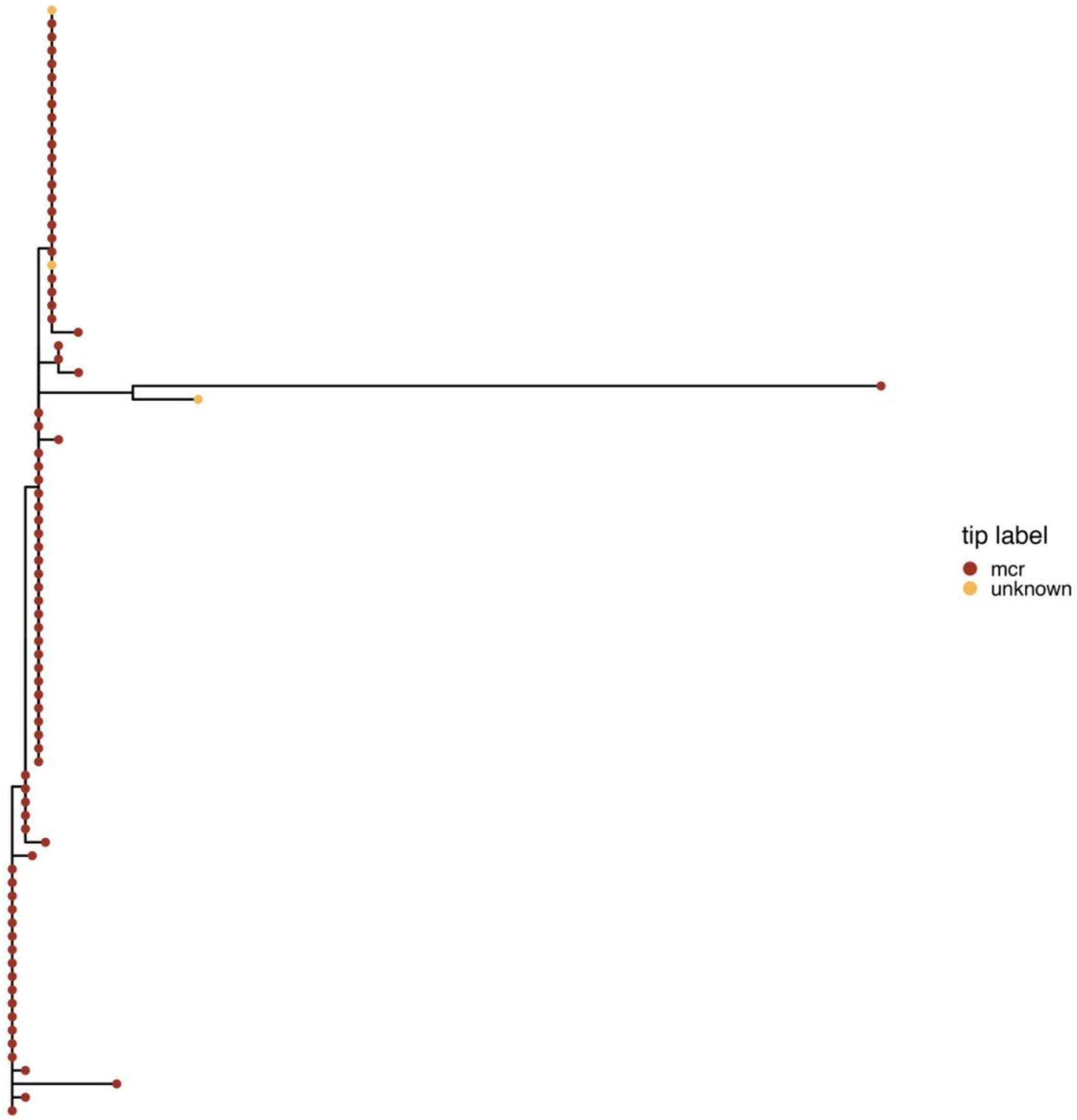

Fig. S4 Phylogenetic tree of *mcr-4* gene.

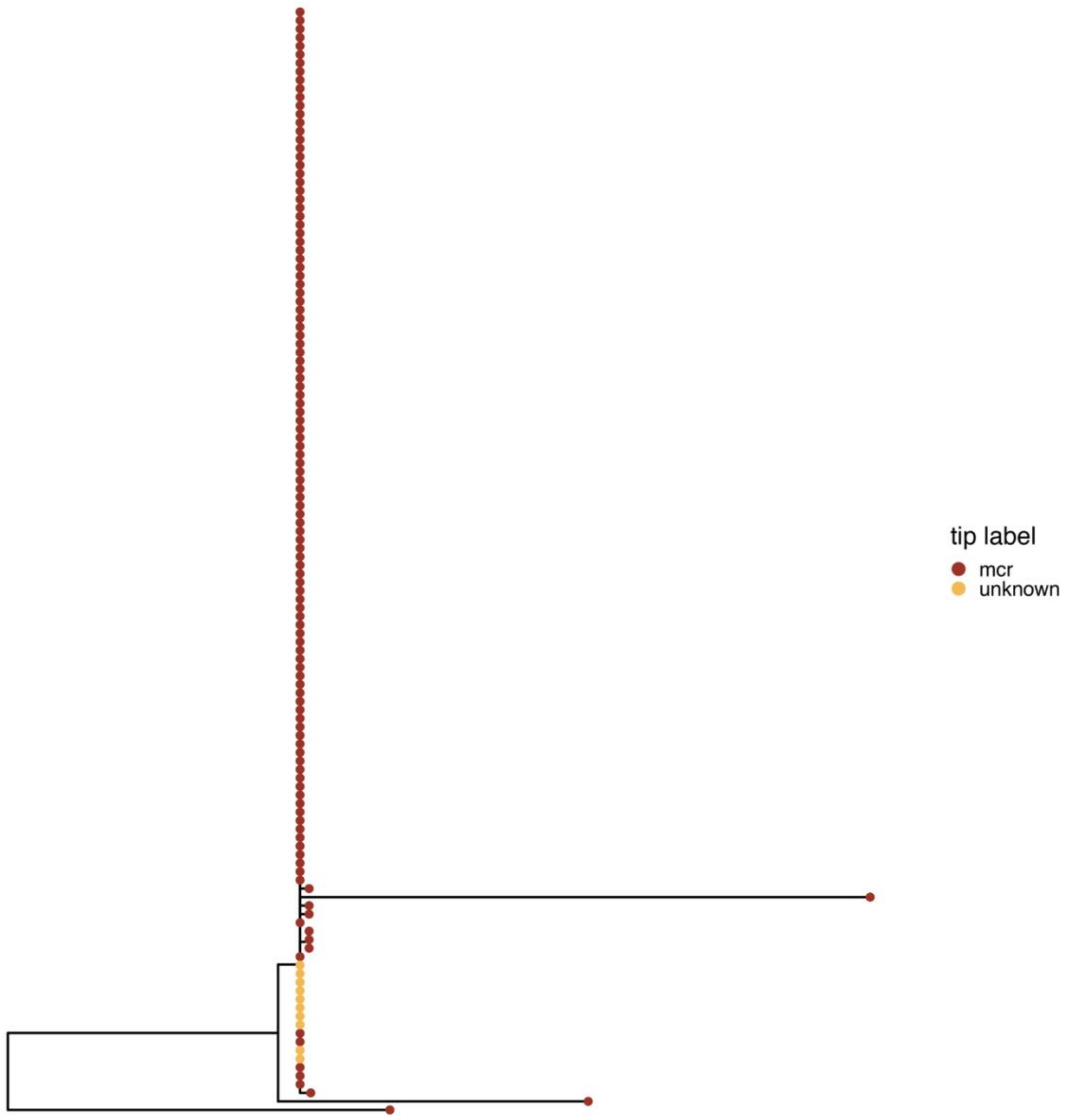

Fig. S5 Phylogenetic tree of *mcr-5* gene.

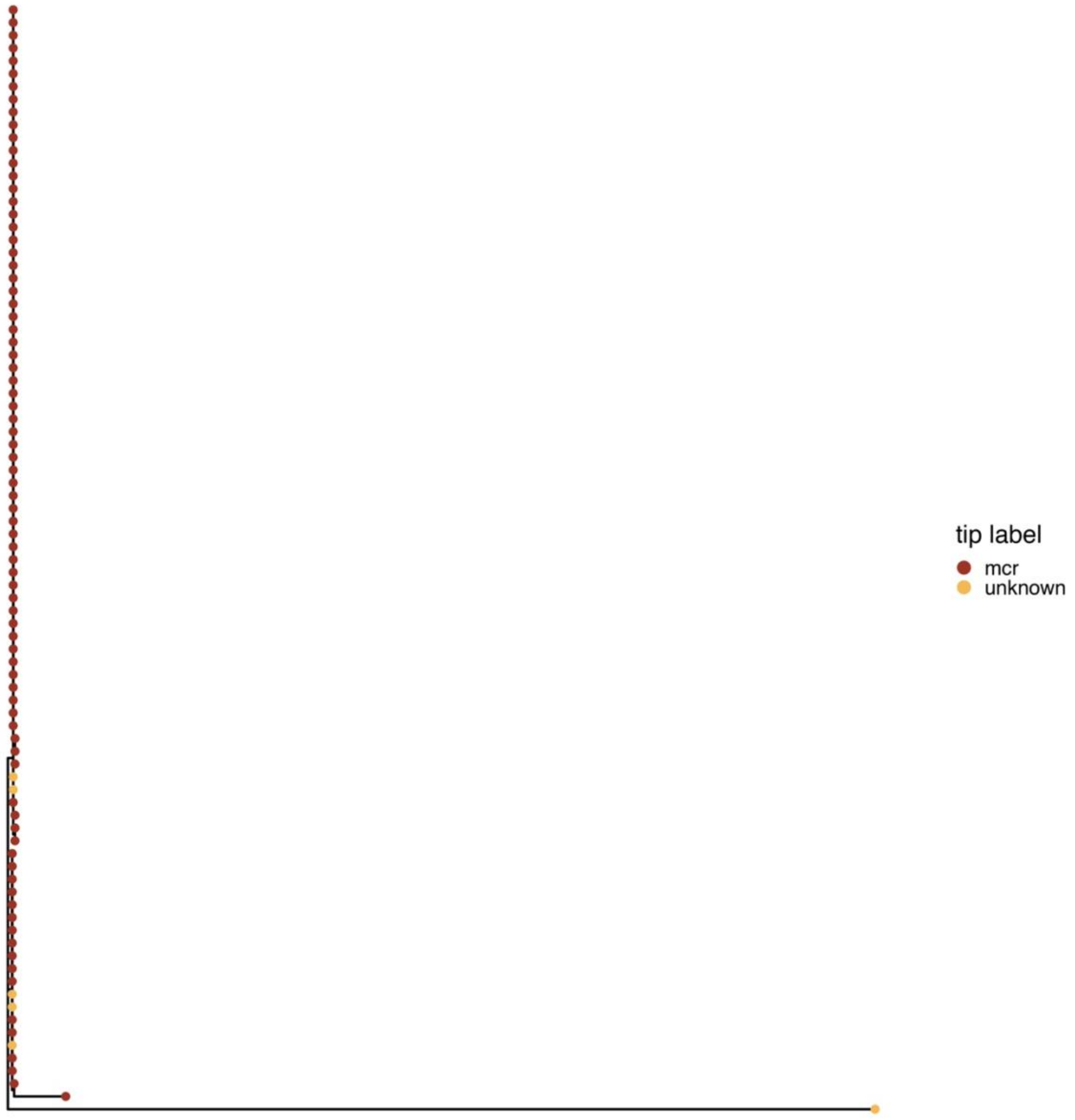

Fig. S6 Phylogenetic tree of *mcr-8* gene.

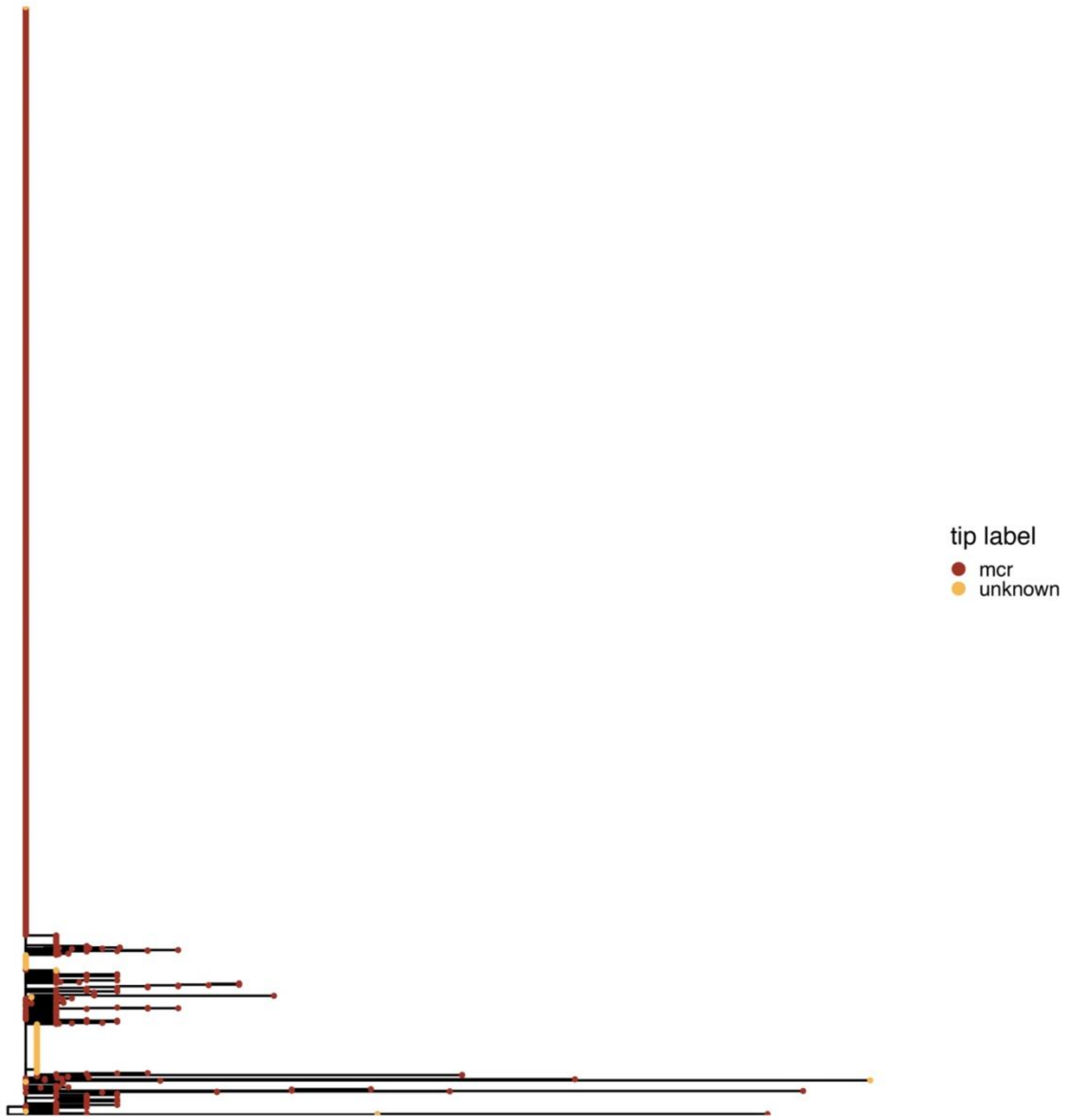

Fig. S7 Phylogenetic tree of *mcr-9* gene.

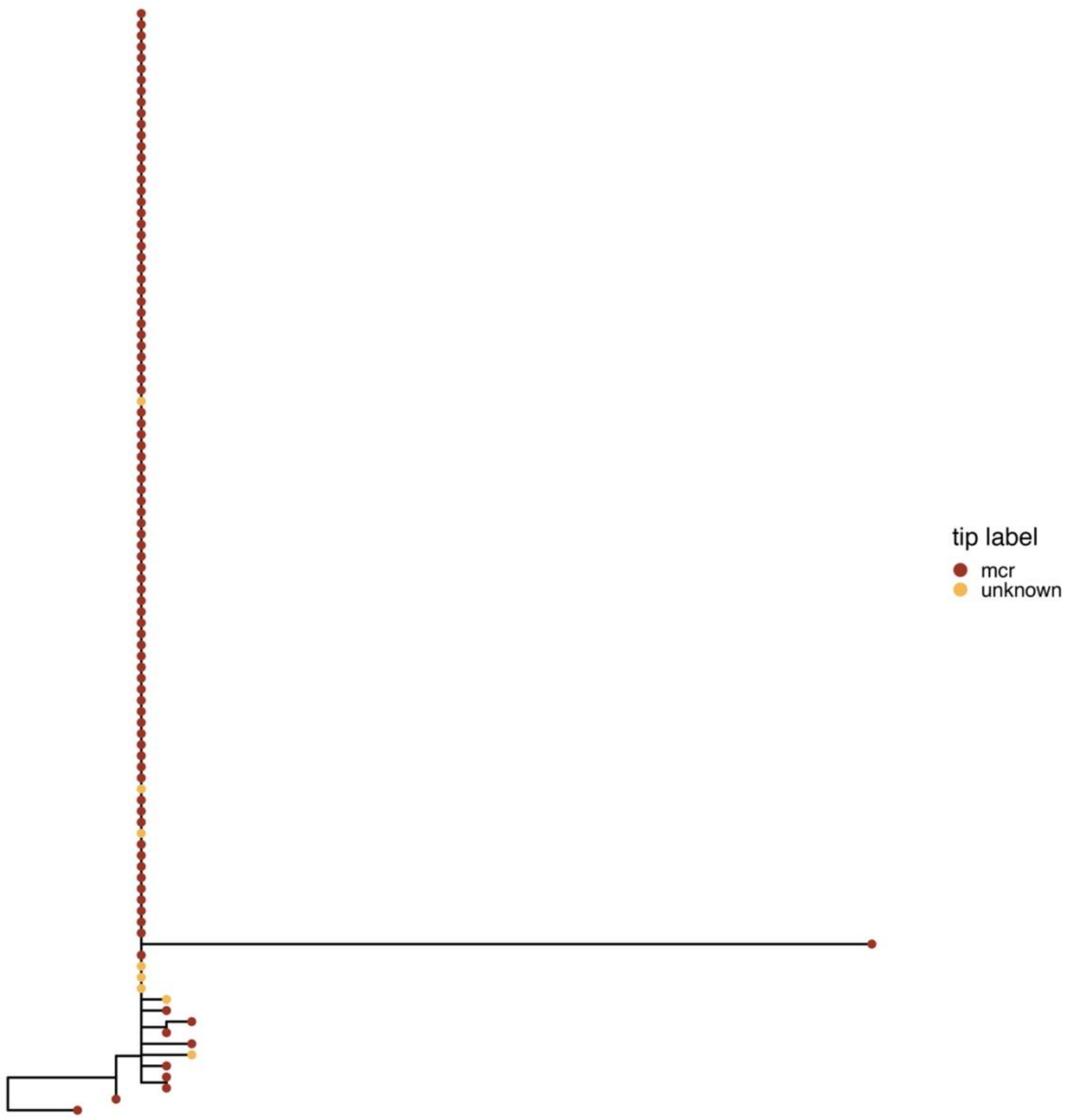

Fig. S8 Phylogenetic tree of *mcr-10* gene.

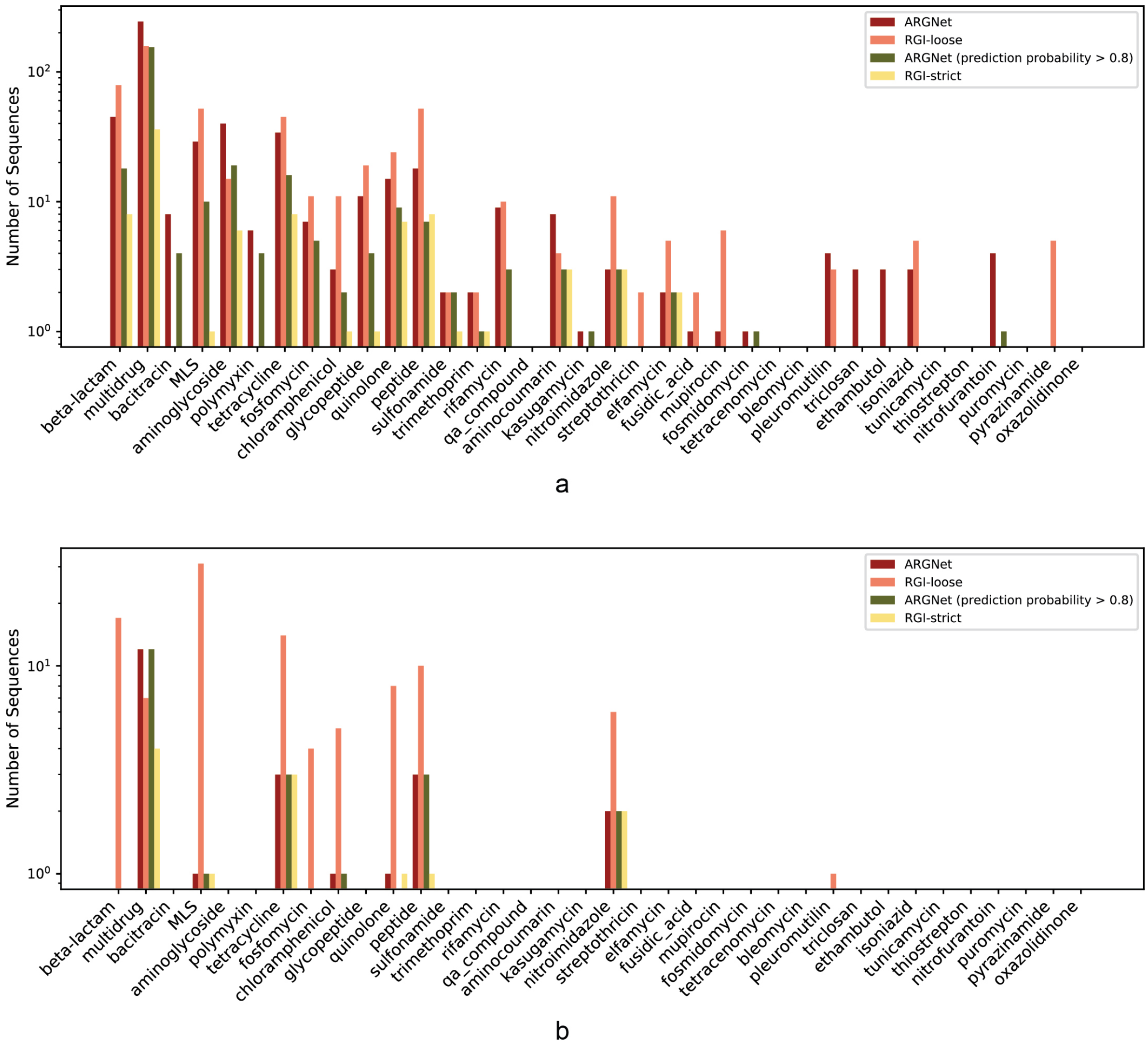

Fig. S9 Prediction on E. coli K12 sequences with ARGNet and RGI. a Classification results of the E. coli K12 sequences identified as ARGs by ARGNet and RGI. **b** Classification results of the transporters sequences from E. coli K12 identified as ARGs by ARGNet and RGI.

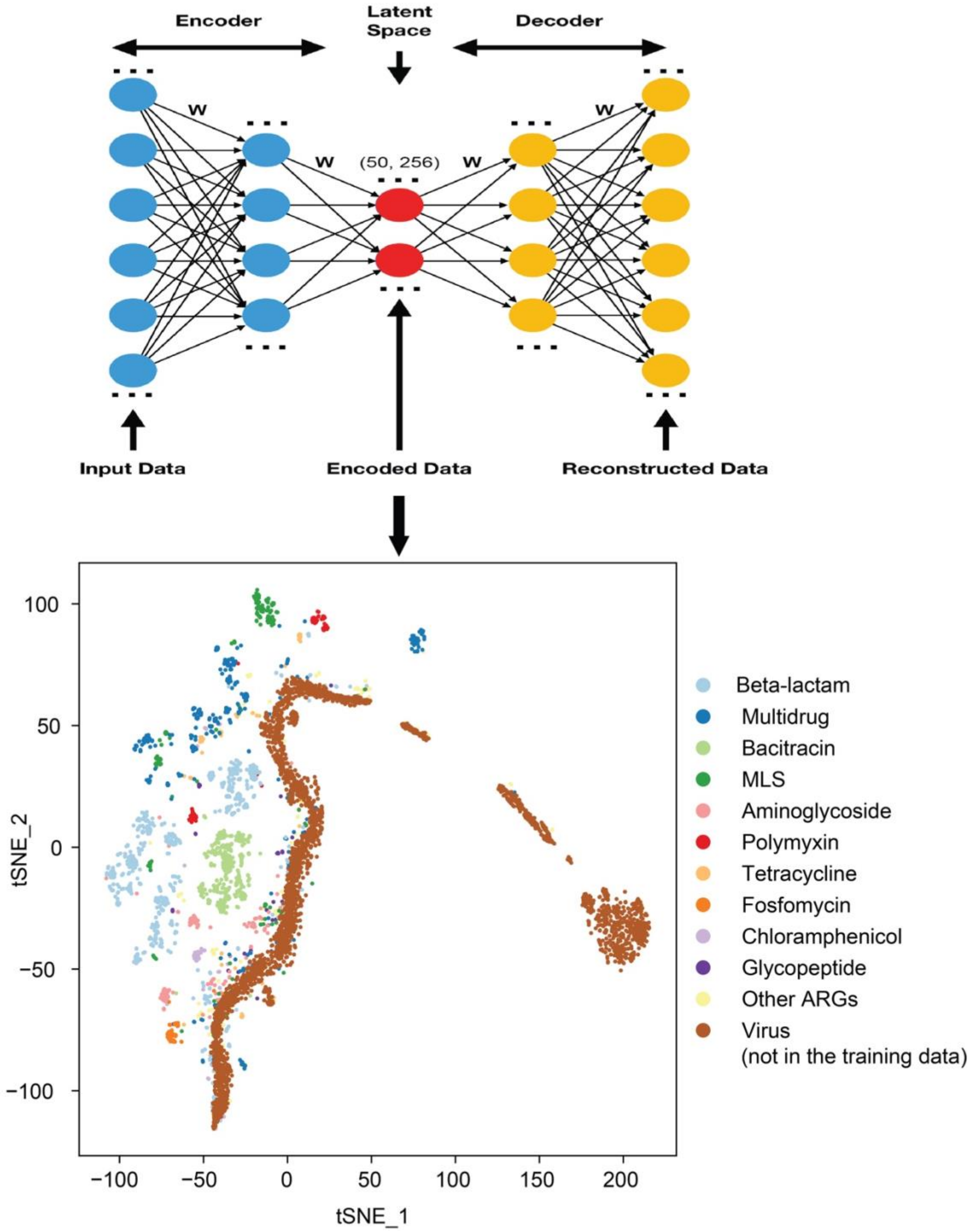

Fig. S10: Latent space representation of autoencoder. The upper part is the illustration of autoencoder model. W denotes the weight of each neuron learned and optimized from the input data. The lower part is dimension reduction via tSNE of the latent space extracted from the ARGNet's autoencoder trained by our ARG reference data (n=21971).

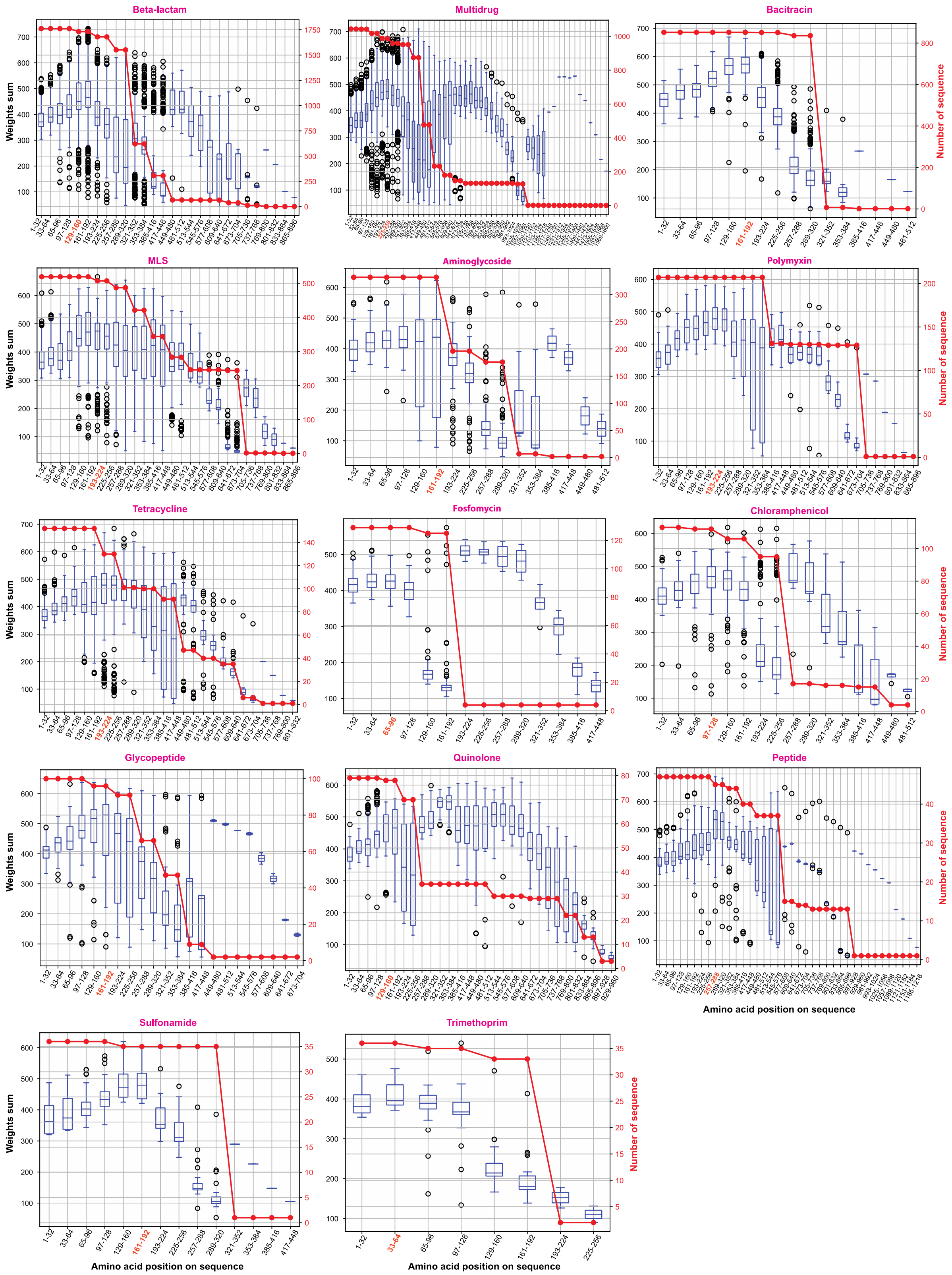

Fig. S11: The weight sum distribution along sequence position in the latent space. Each boxplot represents the weight sum distribution of all test sequences in

each category. The red line chart shows the number of sequences (right y-axis) on each range of positions (x-axis). The highest weight sum position ranges (x-axis) supported by enough number (> 90%) of sequences are highlighted in orange.
